# Proteomics and Machine Learning Approaches Reveal a Set of Prognostic Markers for COVID-19 Severity With Drug Repurposing Potential

**DOI:** 10.3389/fphys.2021.652799

**Published:** 2021-04-27

**Authors:** Kruthi Suvarna, Deeptarup Biswas, Medha Gayathri J. Pai, Arup Acharjee, Renuka Bankar, Viswanthram Palanivel, Akanksha Salkar, Ayushi Verma, Amrita Mukherjee, Manisha Choudhury, Saicharan Ghantasala, Susmita Ghosh, Avinash Singh, Arghya Banerjee, Apoorva Badaya, Surbhi Bihani, Gaurish Loya, Krishi Mantri, Ananya Burli, Jyotirmoy Roy, Alisha Srivastava, Sachee Agrawal, Om Shrivastav, Jayanthi Shastri, Sanjeeva Srivastava

**Affiliations:** ^1^Department of Biosciences and Bioengineering, Indian Institute of Technology Bombay, Mumbai, India; ^2^Centre for Research in Nanotechnology and Sciences, Indian Institute of Technology Bombay, Mumbai, India; ^3^Department of Chemistry, Indian Institute of Technology Bombay, Mumbai, India; ^4^Department of Chemical Engineering, Indian Institute of Technology Bombay, Mumbai, India; ^5^Department of Genetics, University of Delhi, New Delhi, India; ^6^Kasturba Hospital for Infectious Diseases, Mumbai, India

**Keywords:** COVID-19 plasma, host response, mass spectrometry, molecular pathways, prognostic biomarkers, proteomics, drug-repurposing, machine learning

## Abstract

The pestilential pathogen SARS-CoV-2 has led to a seemingly ceaseless pandemic of COVID-19. The healthcare sector is under a tremendous burden, thus necessitating the prognosis of COVID-19 severity. This in-depth study of plasma proteome alteration provides insights into the host physiological response towards the infection and also reveals the potential prognostic markers of the disease. Using label-free quantitative proteomics, we performed deep plasma proteome analysis in a cohort of 71 patients (20 COVID-19 negative, 18 COVID-19 non-severe, and 33 severe) to understand the disease dynamics. Of the 1200 proteins detected in the patient plasma, 38 proteins were identified to be differentially expressed between non-severe and severe groups. The altered plasma proteome revealed significant dysregulation in the pathways related to peptidase activity, regulated exocytosis, blood coagulation, complement activation, leukocyte activation involved in immune response, and response to glucocorticoid biological processes in severe cases of SARS-CoV-2 infection. Furthermore, we employed supervised machine learning (ML) approaches using a linear support vector machine model to identify the classifiers of patients with non-severe and severe COVID-19. The model used a selected panel of 20 proteins and classified the samples based on the severity with a classification accuracy of 0.84. Putative biomarkers such as angiotensinogen and SERPING1 and ML-derived classifiers including the apolipoprotein B, SERPINA3, and fibrinogen gamma chain were validated by targeted mass spectrometry-based multiple reaction monitoring (MRM) assays. We also employed an *in silico* screening approach against the identified target proteins for the therapeutic management of COVID-19. We shortlisted two FDA-approved drugs, namely, selinexor and ponatinib, which showed the potential of being repurposed for COVID-19 therapeutics. Overall, this is the first most comprehensive plasma proteome investigation of COVID-19 patients from the Indian population, and provides a set of potential biomarkers for the disease severity progression and targets for therapeutic interventions.

## Introduction

A previously unknown infectious virus has triggered a raging pandemic of COVID-19 in the year 2020. It has left behind a trail of more than a million dead and destroyed life and livelihood. The viral pathogen that we now know as the Severe Acute Respiratory Syndrome Coronavirus 2 (SARS-CoV-2) (Wu et al., 2020) is that a beta coronavirus belongs to the order Nidovirales of the Coronaviridae family. Thus, the virus has the same lineage as other highly infectious viruses SARS-CoV and Middle East respiratory syndrome coronavirus (MERS-CoV) which has caused outbreaks in the preceding decades (Bradley and Bryan, 2019). In humans, SARS-CoV-2 attaches to the angiotensin-converting enzyme 2 (ACE2) and infects the respiratory tract and lungs, mostly leading to typical flu-like symptoms such as dry cough, body ache, and fever (Li et al., 2020). In some cases, it can also lead to acute respiratory distress syndrome. Patients with such severe symptoms deteriorate leading to multiorgan dysfunction and eventually death (Huang et al., 2020; Zhou et al., 2020) despite intense medical intervention. Although SARS-CoV-2 primarily targets the respiratory tract and lung, several research studies have reported that the virus also infects other organs like the gastrointestinal tract, liver, kidney, cardiac muscles, central nervous system, musculoskeletal system, and even reproductive system in males (Kimhofer et al., 2020; Nie et al., 2020).

The rapid diagnosis of the COVID-19 has been possible due to the increased availability and deployment of RT-PCR assays (Corman et al., 2020), or serological test kits, and scaling-up of testing rates globally (Hosseini et al., 2020). However, the prognosis of the disease remains to be a challenge since the precise pathophysiological pathways that get perturbed during the disease progression remain largely unexplored. Blood is the only body fluid that reaches all organ systems of the body and potentially carries information of physiological perturbations. Further, owing to its minimally invasive nature, it has been the mainstay of several diagnostic tests used for assaying multiple clinical parameters to assess the physiological state of the human body. Indeed, blood is the biofluid of choice to understand physiological aberrances, and thus blood plasma proteome is an excellent source for assessing host response and identifying prognostic markers (Geyer et al., 2017).

The sensitive and high-throughput mass spectrometers have allowed scientists to detect even the faintest of changes in host physiology (Greco et al., 2014). In this study, we employed a deep proteomics strategy to delineate the systems-wide perturbations occurring in non-severe and severe COVID-19 patients. We surveyed the plasma proteome of blood collected from 71 patients of varying disease severity during their active infection phase. Label-free quantification (LFQ) using mass-spectrometry analysis identified around 38 differentially expressed proteins in severe COVID-19 patients when compared with non-severe. These differentially expressed proteins further revealed the dysregulations in the molecular pathways, especially relating to inflammatory pathways, complement activation, and blood clotting.

Machine learning algorithms can parse through voluminous data and pick up a pattern that would otherwise go unnoticed to the human eye. Machine learning (ML) approaches have allowed biologists to uncover the underlying biology of large-scale omics datasets. ML has started to make its presence felt in the field of biomedical sciences, and it has helped scientists understand many human diseases (Ahmed et al., 2020; Alsuliman et al., 2020; Ben-Israel et al., 2020; Hofer et al., 2020; Wilkinson et al., 2020) ranging from cancers (Arif et al., 2020; Chaurasia and Pal, 2020; Jiang et al., 2020) to Ebola (Colubri et al., 2019) as well as to design drugs and study their resistance (Nápoles et al., 2014). ML has also been applied to understand the multifarious aspects of COVID-19 (Elaziz et al., 2020; Lalmuanawma et al., 2020; Tog̃açar et al., 2020; Vaid et al., 2020). We have analyzed our dataset using ML-based techniques to identify clinical classifiers of COVID-19 severity. This set of identified classifier proteins could have a role in disease progression and potentially serve as prognostic biomarkers for the disease.

Further, we have performed targeted mass spectrometry–based multiple reaction monitoring (MRM) analysis to validate our finding from the LFQ analysis. We have also screened a customized drug library against the LFQ identified host proteins using *in silico* docking approaches for COVID-19 therapeutics. The analysis revealed two FDA-approved drugs with the best binding affinity toward the therapeutic targets reported in the study. These results provide valuable information on plasma biomarkers associated with severity of COVID-19 and unravel the mechanistic pathways related to SARS-CoV-2 infection. The potential FDA-approved drugs showing inhibition towards the upregulated marker proteins reported in our study could be ideal for further clinical trials for COVID-19 therapeutics.

## Materials and Methods

### Sample Collection and Clinical Details

For this study, we procured plasma samples from 74 patients who visited Kasturba Hospital for Infectious Diseases, Mumbai, between July and September 2020. All plasma samples were collected with approval from the Institute Ethics Committee, IIT Bombay, and Institutional Review Board, Kasturba Hospital for Infectious Diseases. Based on RT-PCR results, these patients were assigned as COVID-19 positive and COVID-19 negative. Depending on the clinical symptoms, positive patients as advised by clinicians were further grouped into severe (patients with mechanical ventilation and having severe symptoms of acute respiratory distress, bilateral pneumonia) and non-severe (patients having mild symptoms of cough, fever, fatigue, and breathlessness without invasive ventilation). The detailed demographic characteristics for the COVID-19 patients are shown in Supplementary Table 1.

For plasma proteomic analysis of COVID-19 infected patients, 20 negative, 18 non-severe, and 33 severe cases were taken forward (Supplementary Table 2). For the MRM validation experiment, 12 COVID-19 positive patient samples were taken forward. About 2 ml of whole blood was collected for biochemical and serological tests from COVID-19, RT-PCR confirmed, and suspected patients. Whole blood was collected in a sterile Vacutainer by trained medical practitioners under aseptic conditions. After performing biochemical tests, the leftover blood (∼1 ml) was collected and centrifuged immediately at 3,000 rpm for 10 min to separate plasma. The plasma was collected from top of the container to avoid contamination with platelets. The separated plasma was then incubated at 56°C for 30 min for viral inactivation (Hu et al., 2020; Burton et al., 2021) and stored at −80°C in cryovials until further processing. The plasma samples were pooled and aliquoted before storing at −80°C. The aliquots of heat inactivated plasma were then transported at 4°C in random batches to Indian Institute of Technology Bombay for further processing. The samples were processed for proteomics analysis at IIT, Bombay. The details of the standardized blood collection and analytic procedures are provided in the Supplementary Material.

### Proteomics Analysis by LFQ

All plasma samples were depleted using top 12 abundant protein depletion spin column (Pierce) to improve the detectability of low-abundance plasma proteins. Then 15 μl of plasma samples was added into the spin column and incubated for 1 h using a rotating shaker. The samples were eluted by centrifugation at 1,500 × *g* for 2 min. The sample was concentrated by vacuum drying it to 1/4 the initial volume. The depleted plasma sample was taken forward for quantification by Bradford assay, taking BSA as standard. To 30 μg of the depleted plasma sample, 6 M urea was added. Before digestion of the protein, the plasma protein extract was reduced with TCEP (final concentration 20 mM) at 37°C for 1 h and then alkylated with iodoacetamide (final concentration 40 mM) for 30 min under dark condition. The solution was diluted six times with 50 mM ammonium bicarbonate to reduce urea concentration and to adjust the pH. The reduced and alkylated proteins were finally subjected to enzymatic digestion by trypsin at an enzyme/substrate ratio of 1:30 for 16 h at 37°C. The digested peptides were then vacuum dried and reconstituted in 0.1% (v/v) formic acid (FA) for desalting using C18 stage tips. The desalted peptides were further dried and reconstituted in 0.1% (v/v) FA. The peptide concentration was calculated using the Scopes method by measuring O.D. values at 205 nm and 280 nm. Peptides (1 μg) were loaded onto the LC column followed by separation along a LC gradient comprising 80% ACN and 0.1% FA for 120 min at a flow rate of 300 nl/min using easy nano LC 1200 system. The MS analysis was performed using an Orbitrap Fusion Tribrid Mass Spectrometer (Thermo Fischer Scientific). BSA was run at the starting and endpoint of each set of the MS run to check the quality of the instrument. Mass spectrometric data acquisition was done in data-dependent acquisition mode with a mass scan range of 375–1700 *m*/*z* and a mass resolution of 60,000. Mass tolerance was set to 10 ppm, with a dynamic exclusion window of 40 s. Data Dependent MS^*n*^ Scan was performed using high collision–induced dissociation with collision energy mode fixed to 30%. The detector was set to orbitrap in MS 60k and MS/MS 15k and maximum injection time of 30 ms. The detailed parameters for the experiment have been provided in Supplementary Figure 2.

### Proteomics Data Analysis and Machine Learning

The raw datasets were processed with MaxQuant (v1.6.6.0) against the Human Swiss-Prot database (downloaded on 09.07.2020), searched with the built-in Andromeda Search Engine of MaxQuant (Tyanova et al., 2016). Raw files were processed within LFQ parameters setting label-type as “standard” with a multiplicity of 1 and match between runs were selected. The Orbitrap was set to Orbitrap Fusion mode. Trypsin was used for digestion with a maximum missed cleavage of 2. Carbamidomethylation of cysteine (+57.021464 Da) was set as the fixed modification, whereas oxidation of methionine (+15.994915 Da) was set as the variable modification. The false discovery rate was set to 1% for the protein and peptide levels to ensure high protein detection/identification reliability. Decoy mode was set to “reverse,” and the type of identified peptides was set to “unique + razor.”

A sample-wise correlation analysis of 74 samples was performed to understand the data quality. Of these, three samples were removed as they did not meet the standards. Proteomic data of 71 samples were taken forward to perform missing values imputation using the k-Nearest Neighbors (kNN) algorithm in Metaboanalyst (Chong et al., 2018). Statistical analysis and data visualization were carried out in Python and Microsoft Excel. The significant differentially expressed proteins were determined using Welch’s *t*-test where *p* values less than 0.05 were used as a cut-off. The violin plots were made with Log2-transformed data, where the significance level was calculated based on *t*-test independent samples with Bonferroni correction (*p* value annotation legend: ns: 5.00e-02 < *p* ≤ 1.00e + 00; ^∗^: 1.00e-02 < *p* ≤ 5.00e-02; ^∗∗^: 1.00e-03 < *p* ≤ 1.00e-02; ^∗∗∗^: 1.00e-04 < *p* ≤ 1.00e-03; ^****^: *p* ≤ 1.00e-04. Furthermore, partial least squares discriminant analysis (PLS-DA) and principal component analysis (PCA) were performed to understand the perturbation of the sample cohorts. The variable importance in projection (VIP) score depicts the weights of each feature in PLS-DA, which was implemented to perform feature selection. The VIP score threshold was relaxed to 0.8, as the input dataset composed of significantly differentially expressed proteins, and 20 features were selected. We selected support vector machine (SVM) linear model to classify severe patients from non-severe patients using Orange 3.0 after comparing multiple popular ML classification models like Random Forest, Logistic Regression, Naïve Bayes and k-Nearest Neighbor (KNN) algorithm (Demšar et al., 2013). A K-fold cross-validation (*k* = 10) was employed in the evaluation of models’ performance, in which the data were split into k randomly chosen subsets of about equal size. One subset was then used to validate the model trained using the remaining subsets. This process was iterated k times such that each subset was used precisely once toward the validation, and at the end average of all k subsets was taken to evaluate the final model performance score. The SVM linear model performance was evaluated and further visually represented by plotting the ROC-AUC curve, parallel coordinate plot, and confusion matrix using Python and MATLAB tools^1^. Furthermore, potential candidates were individually validated using the SVM linear classification model from a Python scikit-learn library^2^.

### Targeted Proteomics by MRM Assay

The statistically significant and upregulated proteins in COVID-19 severe compared with non-severe obtained from the LFQ data were selected and used for a targeted MRM study. The list of transitions was prepared for unique peptides of these selected proteins using Skyline (version 20.2.1.286). The missed cleavage criterion was 0, and precursor charges +2, +3, and product charges were set at +1 and +2. Further, y ion transitions (from ion 3 to last ion -1) were also included. Pooled samples from both COVID-19 positive and negative patients were run against all the generated transitions and a final list was prepared based on the initial screening. This list consisted of 35 peptides from 13 proteins and included a spiked-in synthetic peptide (FEDGVLDPDYPR) essential for monitoring the consistency of the mass spectrometry runs (with a heavy labeled C-terminal arginine). For the experiment, a Vanquish UHPLC system (Thermo Fisher Scientific, United States) connected to a TSQ Altis mass spectrometer (Thermo Fisher Scientific, United States) was used. The peptides were separated using a Hypersil Gold C18 column 1.9 μm, 100 × 2.1 mm (Thermo Fisher Scientific, United States) at a flow rate of 450 μl/min for a total time of 10 min. A binary buffer system comprising 0.1% FA as the buffer A and 80% ACN in 0.1% FA as buffer B was used. Approximately 1 μg of BSA was also run with the samples to check uniformity in the instrument response.

A batch of samples that included six severe and six non-severe COVID-19 samples were run in duplicates and data were acquired against the aforementioned peptides list containing 36 peptides. The first and second replicates were run 2 days apart to establish reproducibility. After the data were acquired, the raw files were imported into Skyline, and peaks were annotated with the help of a library built from the in-house LFQ data of COVID-19 samples.

### Biological Pathway Analysis and Molecular Docking

The biological pathway analysis was done using Metascape for GO enrichment analysis (Zhou et al., 2019), whereas STRING (version 11.0) (Szklarczyk et al., 2019) was used to prepare the protein–protein interaction network (PPIN). The node colors in the PPIN represent the biological process, whereas the edge width depicts the confidence of the association. The trends of the protein were shown with the help of arrows and the expressional changes of few proteins were supported by violin plots. Differentially expressed proteins from our proteomic study have been taken forward for *in silico* docking studies where we retrieved the complete crystal structures of the proteins available from Protein Data Bank (PDB) (Berman et al., 2002). The known inhibitors were searched in the literature against the selected proteins and termed as control inhibitors. The binding affinity (kcal/mol) was documented for each control. We prepared a library of 58 small molecular components, of which 30 were FDA approved, 9 are in clinical trials, and 19 are in pre-clinical phase trials. Spatial Data File (SDF) for each of the components were downloaded from the ZINC-15 database (Sterling and Irwin, 2015). The proteins had a complete crystal structure, and known inhibitors were taken forward in this study, where each inhibitor was docked against the library along with their respective controls. We used AutoDock Vina 1.1.2 (Trott and Olson, 2019) to perform the docking experiment, which was inbuilt in PyRx software^3^. After loading the.pdb structure of proteins, they were first converted to a macromolecule via AutoDock tools. Similarly, SDF files for the selected drugs were converted to PDBQT format, which is a readable file format for AutoDock Vina, using the open babel tool. In our blind docking method, the exhaustiveness was set to 50 while instead of choosing a particularly active site, the whole protein was contoured into the grid box. The docking output files were split into individual poses where the pose having the lowest binding energy was taken forward for further analysis. Finally, the docked structures were visualized using PyMOL (version 2.4) and Discovery Studio Visualizer Software (version 4.0) and checked for the binding pockets for the drugs in the library. In addition, the protein–ligand interaction profiler (PLIP) server was used to calculate the number and types of interactions between the protein and the drugs (Salentin et al., 2015).

## Results

### Deep Proteomic Analysis of Patient Plasma

**FIGURE 1:**
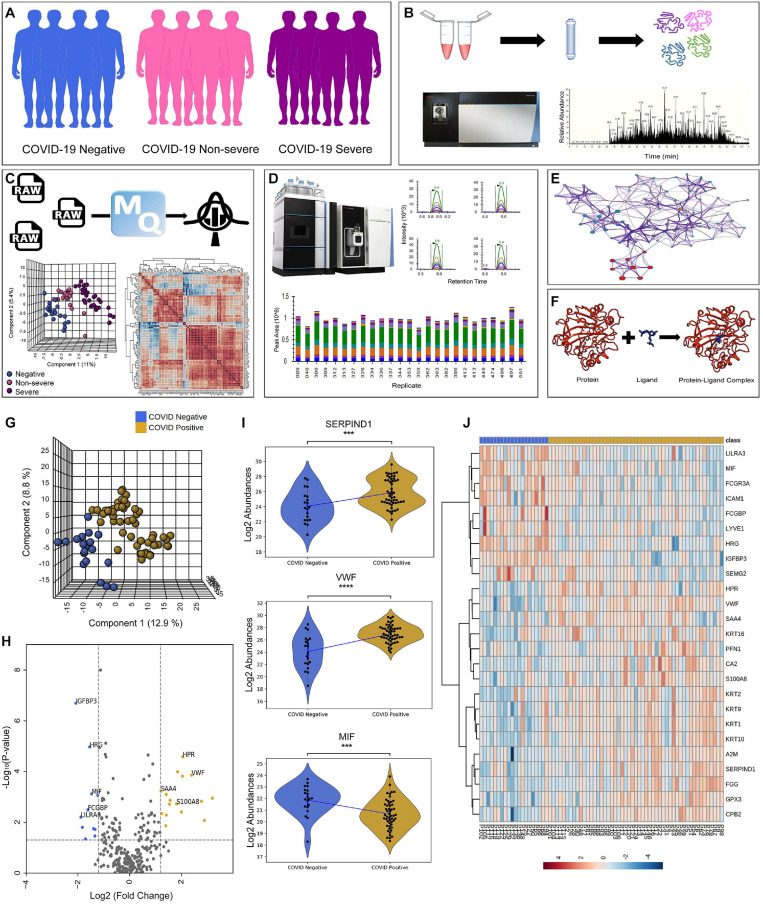
Schematics of plasma proteomics and proteins dysregulated in the COVID-19 positive when compared with negative. **(A)** Sample cohort size consisting of 20 COVID-19 negative, 18 COVID-19 non-severe, and 33 COVID-19 severe patients. **(B)** Schematic workflow of label-free quantification under discovery proteomics. **(C)** Overview of statistical data analysis. **(D)** Workflow of validation using multiple reaction monitoring (MRM) approach showing the representative peaks of synthetic peptides. **(E,F)** Outline of biological network analysis using Metascape and docking study performed using AutoDock Vina, respectively. **(G)** Partial least squares–discriminant analysis (PLS-DA) of 71 patient samples showing the segregation between COVID-19 positive (including severe and non-severe) and COVID-19 negative samples. **(H)** Volcano plot showing significant differentially expressed protein between COVID-19 positive and negative. **(I)** Violin plot of few of the dysregulated host proteins such as SERPIND1, VWF, and MIF protein in COVID-19 positive (***1.00e-04 < *p* ≤ 1.00e-03). **(J)** Heatmap of top 27 significant differentially expressed proteins in COVID-19 positive and negative.

We performed LFQ of a total of 74 depleted plasma samples, of which 20 were negative, 18 were non-severe, and 36 were severe (Figure 1A). Figures 1B–F depicts the schematic workflow of LFQ under discovery proteomics, statistical analysis of data, the summary of synthetic peptide peaks after MRM under validation proteomics, and represents the outline of biological network analysis and docking study, respectively. The correlation matrix of all the 74 samples is shown in Supplementary Figure 1. The mass-spectrometry setting for the LFQ is shown in Supplementary Figure 2. The LFQ analysis of 74 samples provides a total of 1,206 proteins. A list of 278 missing value imputed proteins from 71 samples was taken forward for the partial least squares–discriminant analysis (PLSDA) for an overall assessment of the difference between the COVID-19 positive and COVID-19 negative sample cohort. The two sample cohorts were found segregating in to separate groups as shown in Figure 1G. Figure 1H depicting a volcano plot shows significant differentially expressed proteins between the two cohorts. The statistical analysis between the COVID-19 positive and COVID-19 negative cohort revealed 27 significant differentially expressed proteins shown in form of a heatmap in Figure 1J. Proteins, namely, von Willebrand factor (VWF), haptoglobin-related protein (HPR), glutathione peroxidase 3 (GPX3), alpha-2-macroglobulin (A2M), carbonic anhydrase 2 (CA2), protein S100-A8 (S100A8), carboxypeptidase B2 (CPB2), heparin cofactor 2 (SERPIND1), fibrinogen gamma chain (FGG), profilin-1 (PFN1), and serum amyloid A-4 protein (SAA4), were found to be significantly upregulated in the COVID-19 positive patients, whereas proteins like lymphatic vessel endothelial hyaluronic acid receptor 1 (LYVE1), intercellular adhesion molecule 1 (ICAM1), macrophage migration inhibitory factor (MIF), histidine-rich glycoprotein (HRG), IgGFc-binding protein (FCGBP), immunoglobulin heavy variable 3-15 (IGHV3-15), and insulin-like growth factor-binding protein 3 (IGFBP3) were shown significantly downregulated in the COVID-19 positive patients. The violin plot of few dysregulated proteins SERPIND1, VWF, and MIF protein are shown in Figure 1I. The list of 27 dysregulated proteins is provided in Supplementary Table 3.

**FIGURE 2:**
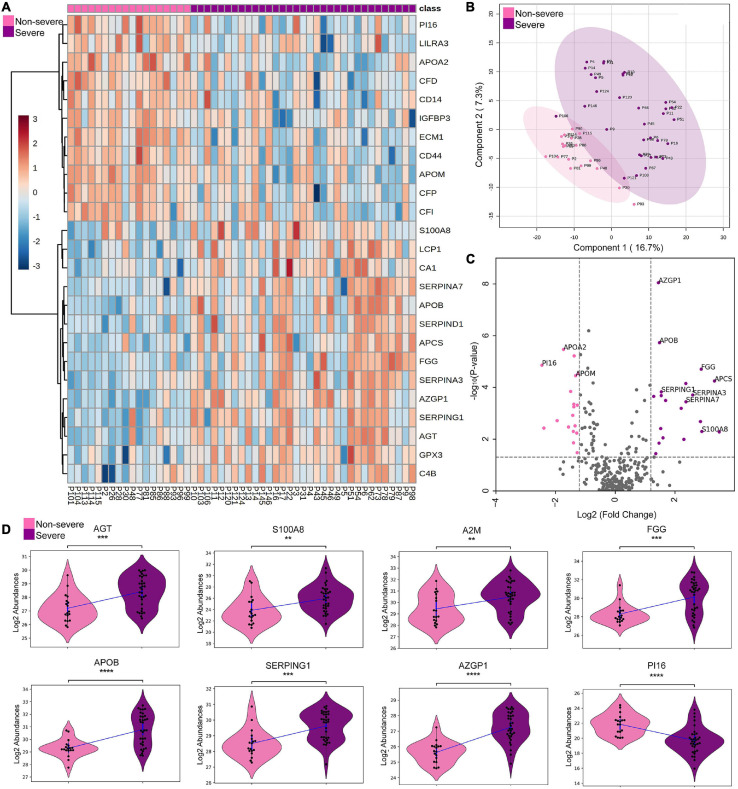
Proteomic analysis of COVID-19 non-severe and COVID-19 severe patients. **(A)** Heatmap of top 25 differentially expressed proteins in COVID-19 severe when compared with the non-severe **(B,C)** depicts the PLS-DA clustering and significant differentially expressed proteins in the form of volcano plot in the COVID-19 severe when compared with the non-severe, respectively. **(D)** Violin plot showing a panel of 8 differentially expressed proteins in the severe vs. non-severe samples (**1.00e-03 < *p* ≤ 1.00e-02; ***1.00e-04 < *p* ≤ 1.00e-03; ****p ≤ 1.00e-04).

Furthermore, this study also investigated the proteomic alterations between the non-severe and severe cohort, which provides a list of 38 significantly differentially expressed proteins (Supplementary Table 4). Figure 2A represents a heatmap of the top 25 differentially expressed proteins in context to the severe and non-severe cohort. A list of 287 missing value imputed proteins was taken forward for the PLS-DA and PCA for an overall assessment of the difference between the severe and non-severe cohort. The two sample cohorts were found segregating into separate groups in PLS-DA with an exception of three samples, P93, P30, and P106, that were observed to be closer to the opposite cluster (Figure 2B). However, 29% cumulative variance of PC1 and PC2 were not able to perturb the severe and non-severe sample cohort completely using PCA (Supplementary Figure 3). Figures 2C,D depicts the significant DEPs in the form of a volcano plot and violin plot respectively. The proteins such as kallistatin (SERPINA4), serum amyloid P-component (APCS), protein S100-A8 (S100A8), fibrinogen gamma chain (FGG), corticosteroid-binding globulin (SERPINA6), and alpha-1-antichymotrypsin (SERPINA3) were found to be upregulated in the severe cohort whereas proteins such as complement factor D (CFD), monocyte differentiation antigen (CD14), complement component C8 alpha chain (C8A), apolipoprotein (LPA), and apolipoprotein M (APOM) were found to be downregulated in severe when compared with non-severe patients. Supplementary Figure 4 represents the top 25 differentially expressed proteins of severe and negative in the form of heatmap and depicts the PLS-DA clustering of the severe versus negative patients.

### Selection of Proteomic Markers Using ML

**FIGURE 3:**
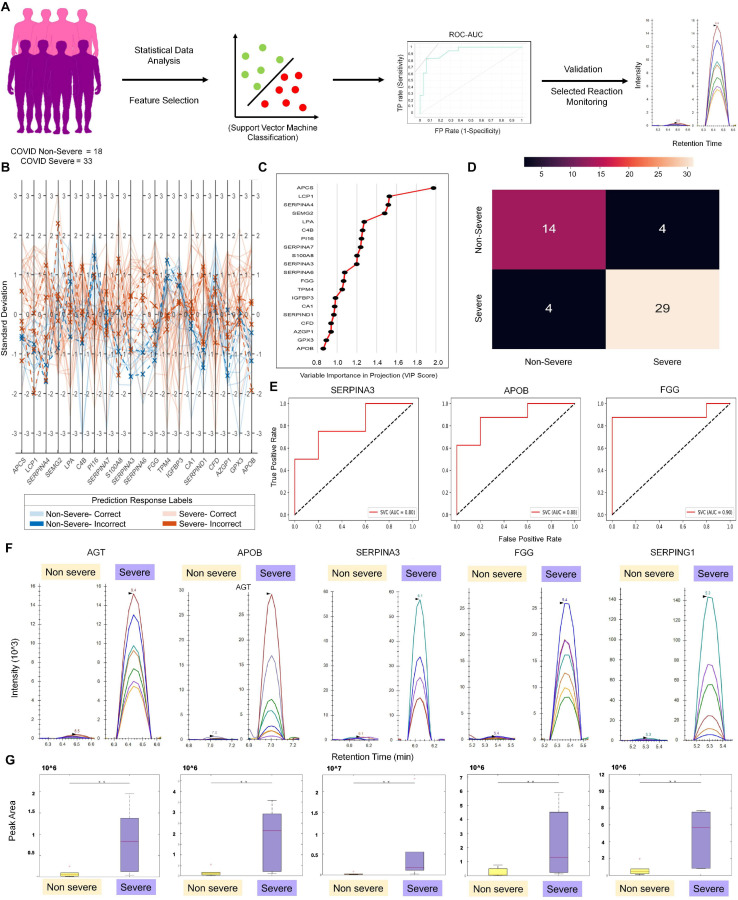
Machine learning–based approach for identification of severity classifiers and validation of protein markers using MRM approach. **(A)** Demonstrates the schematic workflow of machine learning and MRM validation. **(B)** The parallel coordinate plot and prediction response labels. **(C)** Depicts the top 20 features and their variable importance in projection (VIP) scores in the *X*-axis. **(D)** The confusion matrix plotted from the model prediction. **(E)** Displays the ROC–AUC curve of SERPINA3, APOB, and FGG from the severity model prediction. **(F)** MRM analysis of proteins overexpressed in COVID-19 severe vs. COVID-19 non-severe patient samples, as identified in the LFQ analysis and machine learning approach. Peak shapes (as seen in Skyline) of representative peptides of proteins AGT, APOB, SERPINA3, FGG, and SERPING1, respectively. **(G)** Box plots showing the overexpression of same proteins in severe as compared with non-severe samples in terms of the MRM peak areas (*t*-test, **p < 0.05; fold change > 3 at a CI of 95–99% determined by Skyline).

A performance metrics of SVM, Logistic Regression, Naïve Bayes, Random Forest, and k-Nearest Neighbor algorithms were compared on the test dataset (Supplementary Table 5). The SVM linear showed the maximum classification accuracy of 0.88 taking 38 DEPs in context to the severe and non-severe cohort (Figure 3A and Supplementary Figure 5). A list of 20 features were selected using VIP score based on PLS-DA. These 20 selected features were further used to build ML models to check and compare the model performance score with the one when the input dataset was 38 significantly differentially expressed proteins. Here also, SVM linear was giving the highest accuracy score among other models like Random Forest, Logistic Regression, Naïve Bayes, and k-Nearest Neighbor. The SVM linear model performance was evaluated and further visually represented by plotting the ROC–AUC curve, parallel coordinate plot, and confusion matrix (Figures 3B,C). The confusion matrix from model prediction showed that 43 samples out of 51 samples were correctly classified (Figure 3D). The performance measurement of the classification from the model prediction depicted AUC more than 0.9 whereas the classification accuracy, precision, F1, and recall was found to be more than 0.84. Proteins like AGT, APOB, SERPINA3, FGG, and SEPRING1 were further taken forward for MRM. Of these, APOB, SERPINA3, and FGG showed accuracy score of linear SVM model more than 0.8 in classification of severe and non-severe samples (Figure 3E).

### MRM Analysis of Proteins Overexpressed in Severe COVID-19

The MRM study aimed to validate the differentially regulated proteins found between COVID-19 severe and non-severe samples from the LFQ data. The response for BSA as QC standard to monitor day-wise instrument performance is shown in Supplementary Figure 6. To establish that all the injections gave the same response, we spiked in an equal amount of a heavy labeled synthetic peptide (FEDGVLDPDYPR) in equal amount in all samples. The uniform peak areas for this peptide, as shown in Supplementary Figure 7, establish the same. Even duplicates run on separate days showed comparable peak areas with low cv. Based on the response of the differentially regulated peptides, the list was further refined to keep only peptides showing significant dysregulation (adjusted *p* values below 0.05) between severe and non-severe. For this, the peaks were annotated, and transitions were refined according to the library match to give dot *p* values for all peptides. A dot *p* value is a measure of the match between the experimental peak and the library fragmentation patterns. Thus, the refined list had 183 transitions belonging to 28 peptides from 9 host proteins and 1 synthetic peptide. The transition of proteins and peptides that exhibited differential regulation between COVID-19 non-severe and severe patient samples are shown in Supplementary Table 6. Using the MSstats external tools in Skyline, we determined that proteins AGT, APOB, SERPINA3, FGG, and SEPRING1 have three or more than three peptides with a peak area fold change of more than 3 and adjusted *p* value less than 0.05 at a confidence of 95–99% (Figure 3F, G). This validates that for the given set of samples, these proteins show statistically significant overexpression in COVID-19 severe patients than in COVID-19 non-severe patients (refer to the data availability section for the Skyline files).

### Biological Pathway and Network Analysis of Differentially Expressed Protein in Severe Versus Non-severe Comparison

**FIGURE 4:**
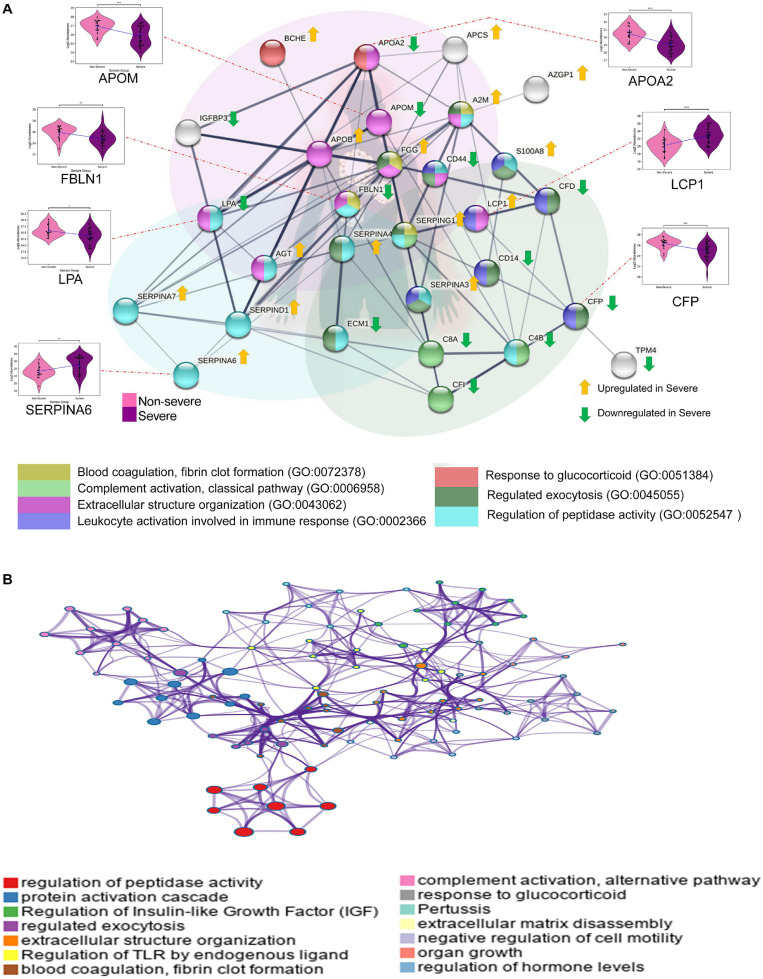
Biological pathways and network analysis of differentially expressed proteins in severe vs. non-severe comparison. **(A)** Represents the Metascape enriched biological processes with their co-expressed proteins in the form a bipartite network where few proteins have been shown in the form violin plot (ns: 5.00e-02 < *p* ≤ 1.00e + 00; *1.00e-02 < *p* ≤ 5.00e-02; **1.00e-03 < *p* ≤ 1.00e-02; ***1.00e-04 < *p* ≤ 1.00e-03; ****p ≤ 1.00e-04). **(B)** The network of enriched terms, generated using String (version 11.0) shows colored clusters, where the nodes that share the same clusters are typically close to each other.

We also identified the enriched biological processes for the 38 dysregulated proteins in COVID-19 severe compared with COVID-19 non-severe patients. The proteins mapping to the enriched biological processes were shown in the form of protein–protein interaction. Few proteins have been shown in the form of a violin plot (Figure 4A). Figure 4B illustrates a network of enriched terms colored by clusters, where nodes that share the same clusters are typically close to each other. We identified biological processes such as regulation of peptidase activity, regulated exocytosis, extracellular structure organization, blood coagulation, fibrin clot formation, complement activation, classical pathway, leukocyte activation involved in immune response, and response to glucocorticoid process to be enriched in COVID-19 severe patients. The list of proteins expressed in these pathways is shown in Supplementary Table 7.

### *In silico* Screening of Drugs Against Differentially Expressed Proteins

We performed *in silico* molecular docking of significantly altered proteins with a library of 58 drugs (Supplementary Table 8), among which 30 are FDA approved, 9 are clinically approved, and 19 are pre-clinical approved. We identified known inhibitors of those altered proteins from the literature and used as positive control drugs for each protein. Positive control drug was used to derive a possible cut-off for the docking score. After docking, the selection of potential drug was based on two major criteria. First, the binding energy of the drug should be equal to or higher than that of the control inhibitor; and second, the binding pocket of the drug should be similar to that of the control drug. Here, we selected five significant proteins for COVID-19 non-severe versus severe comparison: heparin cofactor 2 (SERPIND1), thyroxine-binding globulin (SERPINA7), angiotensinogen (AGT), carbonic anhydrase-1 (CA1), and carbonic anhydrase-2 (CA2) (Supplementary Table 9) for docking study. The list of potential drugs binding to these target proteins is provided in Supplementary Tables 10, 11.

**FIGURE 5:**
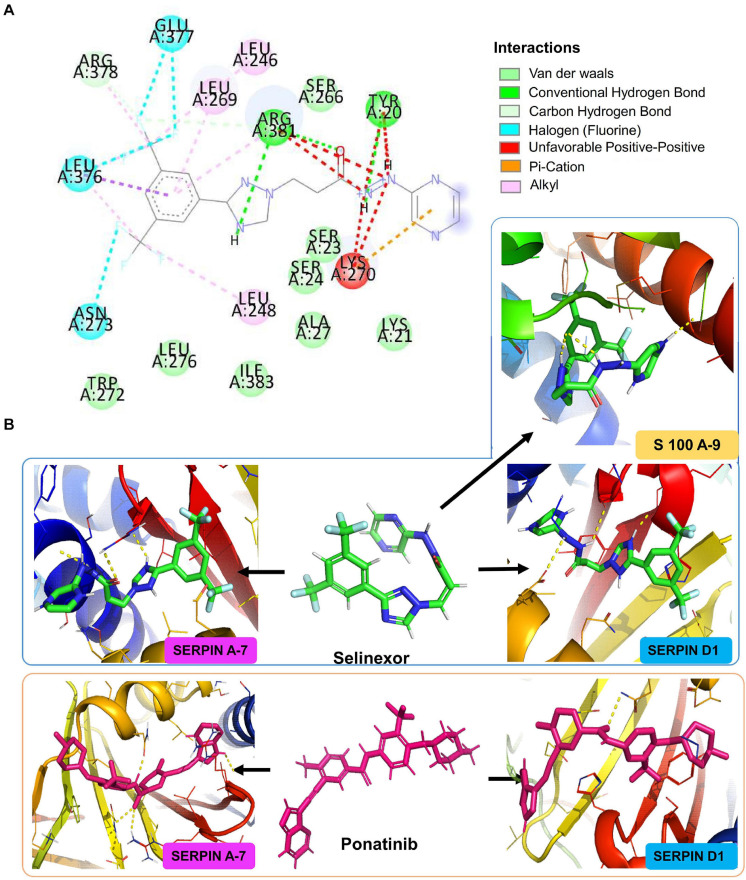
*In silico* molecular docking of small molecules against upregulated proteins from different stages of COVID-19 infection. Figure showing the docking results of three different target proteins SERPINA7 (non-severe vs. severe), SERPIND1 (non-severe vs. severe), and S100A9 (COVID-19 positive vs. COVID-19 negative) with two FDA-approved drugs selinexor and ponatinib. **(A)** The predicted 2D interaction map of selinexor with SERPINA7. **(B)** The 3D representation of predicted binding pockets of the two drugs to the targeted proteins. The binding affinity of selinexor SERPINA7 is -9.3 kcal/mol, SERPIND1 is -8.7 kcal/mol of binding affinity, and S100A9 is -7.5 kcal/mol. Drug ponatinib binds with SERPINA7 with -9.3 kcal/mol of binding affinity and with SERPIND1 with -8.4 kcal/mol binding affinity.

Heparin cofactor 2 (SERPIND1) protein composed of 499 amino acid long peptide was shown to bind with the drug sulodexide with a binding affinity of -7.1 kcal/mol and hence taken as a control drug (Supplementary Figure 8A). When docked with the customized drug library, four FDA-approved drugs were found that to have a similar binding pocket as that of the control drug and have better binding affinity than sulodexide, namely, selinexor (-8.7 kcal/mol), ponatinib (-8.4 kcal/mol), epigallocatechin gallate or EGCG (-7.7 kcal/mol), and nafamostat (-8.1 kcal/mol). Similarly, selinexor and ponatinib also exhibited a higher binding affinity for thyroxin-binding globulin or SERPINA7, a protein with 415 amino acids. Tamoxifen, the control inhibitor of SERPINA7, showed a binding affinity of -7.4 kcal/mol (Supplementary Figure 8B). A conventional hydrogen bond formation between the selinexor and Y20 and R381 amino acid of SERPINA7 was visualized at two-dimensional plane using Discovery Studio Visualization software (Figure 5A). Selinexor and ponatinib had a binding affinity of -9.3 kcal/mol for the protein (Figure 5B). Angiotensinogen (AGT), a 485 amino acid long protein, had a binding affinity of -8.4 kcal/mol for irbesartan which was used as a control drug in our study (Supplementary Figure 8C). It also exhibited a binding affinity of -8.9 kcal/mol for ML-240 which is a pre-clinical approved drug. In our study, ML-240 was found to be the most potential drug to target AGT. We also performed the molecular docking of carbonic anhydrase-1 (CA1) (261 amino acid length) and carbonic anhydrase-2 (CA2) (260 amino acid length) with the drug library. The binding affinity of small molecule topiramate for CA1 was -9.2 kcal/mol (Supplementary Figure 8D) while acetazolamide was shown to bind CA2 with a binding affinity of -6.3 kcal/mol (Supplementary Figure 8E), hence were used as control drug for respective proteins. Our study identified EGCG as the only FDA-approved drug that can be used to target CA1 (with binding affinity -9.5 kcal/mol) and nafamostat (with binding affinity -8.2 kcal/mol) to target CA2. Similarly, four significantly dysregulated proteins from COVID-19 positive versus negative comparison were chosen for molecular docking. These included protein S100A9 (S100A9), carboxypeptidase B2 (CPB2), glutathione S-transferase omega-1 (GSTO1), and 6-phosphogluconate dehydrogenase (6-PGDH1). The list of potential drug binding to these target proteins is provided in Supplementary Tables 10, 12.

Rapamycin is a known mTOR inhibitor. In our study, rapamycin drug was found to be the most potential drug to target all of these four proteins pertaining to their higher binding affinity and competitive binding for the targets as compared with control drugs. The first protein was S100A9, which is a small protein with 114 amino acids. We used tasquinimod as a control inhibitor as it interacts with the protein with a binding affinity of -7.5 kcal/mol (Supplementary Figure 8F). Using the criteria mentioned previously for selection drugs, we identified S100A9 can be targeted using two FDA-approved drugs: 1) selinexor, which had a binding affinity of -7.5 kcal/mol, and 2) rapamycin with a binding affinity of -8.2 kcal/mol.

Carboxypeptidase B2 (CPB2) is a 423 amino acid long protein. We used anabaenopeptin F as a control drug because its binding affinity for CPB2 was -8.3 kcal/mol (Supplementary Figure 8G). Here, we identified three FDA-approved drugs rapamycin (binding affinity -8.7 kcal/mol), dabrafenib (binding affinity -8.8 kcal/mol), and daunorubicin (binding affinity -8.6 kcal/mol), which can target CPB2.

CMFDA is reported to be a known inhibitor of glutathione S-transferase omega-1 (GSTO1) protein and it binds to the protein with a binding affinity of -8.3 kcal/mol (Supplementary Figure 8H). Our study showed that four FDA-approved drugs from our customized drug library have the potential to target GSTO1, which are rapamycin (binding affinity -8.8 kcal/mol), selinexor (binding affinity -8.6 kcal/mol), silmitasertib (binding affinity -8.3 kcal/mol), and ponatinib with highest binding affinity of -9.1 kcal/mol. Physcion was another known inhibitor of 6-phosphogluconate dehydrogenase (6-PGDH1) used as a control drug as it was found to bind 6-PGDH1 with a binding affinity of -7.0 kcal/mol (Supplementary Figure 8I). Six FDA-approved drugs from our customized drug library can be used to target 6-PGDH1. These include rapamycin (binding affinity -8.8 kcal/mol), selinexor (binding affinity -8.5 kcal/mol), ponatinib (binding affinity -10.3 kcal/mol), silmitasertib (binding affinity -7.7 kcal/mol), daunorubicin (binding affinity -8.4 kcal/mol), and dabrafenib (binding affinity -8.6 kcal/mol). Rapamycin, an already approved drug for organ transplant rejection, was observed to bind to all four proteins significantly upregulated in COVID-19 positive patients. We also found that selinexor, an exportin antagonist, and ponatinib, a tyrosine kinase inhibitor, approved for use in treatment of multiple myeloma and chronic myeloid leukemia (CML), respectively, can be used to target proteins altered in severe cases as compared with non-severe.

## Discussion

Nasopharyngeal swab samples and serological tests are being routinely used in clinics for the diagnosis of SARS-CoV-2 infection. However, biomarkers for prognosis of the disease before it could lead to fatality are yet to be found. Understanding the host response toward the viral infection might provide important clues on the disease progression from non-severe to severe. A mass spectrometry proteomics approach was applied for a granular understanding of the disease mechanism. Studies have already reported differences in the level of blood-based proteins such as lactate dehydrogenase (LDH), D-dimers, and inflammatory markers such as C-reactive protein (CRP), ferritin, and fibrinogen in COVID-19 patients (Lagadinou et al., 2020; Zhu et al., 2020). One specific forte of our study is the in-depth profiling of plasma proteome from a cohort of COVID-19 patients (*n* = 71), facilitating the robust and statistically significant evaluation of differential expression between non-severe and severe disease groups. Our study holds importance in understanding the biology associated with COVID-19 progression as the Indian subcontinent seems reasonably unscathed by the pandemic with the fatality rates being among the lowest in the world (Dong et al., 2020).

Deep proteome analysis of COVID-19 plasma samples revealed a subset of proteins that were significantly dysregulated in the positive samples when compared with the negative controls. Proteins namely von Willebrand factor (VWF), haptoglobin-related protein (HPR), glutathione peroxidase 3 (GPX3), alpha-2-macroglobulin (A2M), carbonic anhydrase 2 (CA2), protein S100-A8 (S100A8), carboxypeptidase B2 (CPB2), heparin cofactor 2 (SERPIND1), fibrinogen gamma chain (FGG), profilin-1 (PFN1), and serum amyloid A-4 protein (SAA4) were found to be significantly upregulated in the COVID-19 positive patients. Previously, animal studies have shown that increased VWF might be due to hypoxic conditions in the lung endothelial cells (Mojiri et al., 2013). However, this induces a risk of arterial or venous thrombosis because it directly promotes the thrombotic process during inflammation (Kawecki et al., 2017). The increase in HPR is also found in cases of idiopathic pulmonary fibrosis (Saraswat et al., 2020) and as a factor of non-bacterial pneumonia (Yang et al., 2018), thus may act as a biomarker of lung trauma. Carboxypeptidase B2 has anti-inflammatory and anti-fibrinolytic effects. Its increase in this cohort indicates the natural response to systemic inflammation brought about by COVID-19 (Tawara et al., 2016). Another protein, PFN1 overexpression, implicated in vascular hyperpermeability and vascular hypertrophy, can perhaps explain the aberrant physiology of COVID-19 patients. The acute phase response proteins such as SAA-4 and S100A8 were also upregulated in response to COVID-19. Interestingly, the protein lymphatic vessel endothelial hyaluronic acid receptor 1 (LYVE1) was downregulated and might indicate liver injury (Arimoto et al., 2010). At the same time, attenuated histidine-rich glycoprotein (HRG) expression might explain the altered hemostasis in the patients (Tsuchida-Straeten et al., 2005). However, there lies a caveat; most of these patients were under medications; the results might also be due to the ongoing therapies than the disease itself.

Further, the deep plasma proteome study of non-severe versus severe COVID-19 patients revealed only 38 differentially expressed proteins, out of which proteins such as FGG, S100A8, VWF, SAA4, SERPIND1, and SERPINA6 were identified to be significantly upregulated in the COVID-19 severe patients. Interestingly, the mitochondrial 60-kDa heat shock protein (HSPD1) was found to be highly expressed in severe patients. It has been already reported that high levels of circulatory HSPD1 are associated with cardiac failures (Sidorik et al., 2005). Therefore, increased HSPD1 in severe patients can act as a potential clinical biomarker of cardiac malfunction in the severe group of patients. Also, our results indicated an increase in plasma cholinesterase (BCHE) in the severe group, which is upregulated in patients suffering from mild ischemic stroke (Assayag et al., 2010). These findings thus implicate severe COVID-19 associated risk of cardiac and CNS injury that has been already reported by clinicians, and these proteins might be potential biomarkers for prognosis.

The plasma levels of carbonic anhydrase 1 (CA1) were found to be substantially elevated in the severe group. Increased carbonic anhydrase has been found to mediate hemorrhagic retinal and cerebral vascular permeability (Gao et al., 2007). The ramifications of increased CA1 are also substantiated by earlier reports on a cohort study of sepsis secondary to pneumonia (Leite et al., 2019), where it was found to be upregulated during sepsis. Moreover, the role of increased CA1 in the worsening of ischemic diabetic cardiomyopathy also paints a rather gloomy picture of the cardiac sequelae of COVID-19, especially in diabetic patients (Torella et al., 2014), and might also contribute to the increased fatality of diabetic patients (Zaki et al., 2020).

The protein fibrinogen (FGG) was also upregulated in severe patients compared with non-severe. FGG is an oligomeric glycoprotein produced in the liver and secreted in the blood. The increased fibrin formation and breakdown correlated with the high level of D-dimers observed in the COVID-19 patients with the worst outcomes (Tang et al., 2020). The increasing level of FGG in severe cases might be due to liver injury, impairing hepatic fibrinogen secretion with acquired fibrinogen storage disease (Fraga et al., 2020). The protein S100A8 (calgranulin A/myeloid-related protein 8) belongs to the group of alarmins or damage-associated molecular patterns (DAMPs), released in response to stress against the microbial infection that leads to exacerbation of the inflammatory response. Chen and his co-workers reported that the level of S100A8 positively correlated with the C_*t*_ value and oxygen demand, indicating the severity of the acute respiratory distress in COVID-19 patients (Chen L. et al., 2020). A recent study showed that severe COVID-19 patients release massive amounts of S100A8, accompanied by changes in monocytes and neutrophil subsets (Silvin et al., 2020). The protein AGT was found to be significantly upregulated in severe patients as compared with the non-severe. Angiotensinogen (AGT) is a component of the renin–angiotensin system (RAS), a substrate of renin that regulates blood pressure and fluid balance. The dysregulation of AGT and RAS might lead to acute lung injury and acute respiratory distress leading to a severe prognosis (Gao Y.L. et al., 2020). Apolipoprotein B-100 (APOB) is involved in lipid transport and low-density lipoprotein (LDL) catabolism.

The high levels of APOB in the plasma might indicate significant cardiovascular manifestation seen in COVID-19 infected severe patients (Moriarty et al., 2020). These results are consistent with previous findings (Shen et al., 2020) on COVID-19 patient sera, which had identified dysregulation of multiple apolipoproteins. Several serine protease inhibitors (SERPINs) such as SERPING1 and SERPINA3 were also identified to be upregulated in severe patients. The increasing level of SERPINs which are acute-phase proteins positively correlates and associates with a high level of IL-6 seen in severe patients (D’alessandro et al., 2020). The validation study using MRM could specifically detect AGT, FGG, APOB, SERPING1, and SERPINA3 host peptides in COVID-19 severe patients. Thus, mass spectrometry–based detection of host peptides has the potential to be used in clinics for the prognosis of disease severity.

A subset of proteins was also downregulated in the severe group. The protein peptidase inhibitor 16 (PI16) was severely downregulated (FC = −2.45, *p* < 0.005). It concurs well with the previous studies. It has been shown that while PI16 plays a protective role against atherosclerosis (Hazell et al., 2016), these results demonstrate the pathogenesis of cardiac maladies in severe COVID-19 patients (Nishiga et al., 2020). Patients with severe COVID-19 often report a lower platelet count (Terpos et al., 2020). Our studies have demonstrated that a crucial factor in platelet biogenesis TPM4 (Pleines et al., 2017) is inhibited (FC-1.94, *p* < 0.001) in severe cases, thereby providing novel biological insight into COVID-19 severity. Another ubiquitously present protein βII spectrin was found to be downregulated in severe COVID-19. Given that inadequate βII spectrin might precipitate into arrhythmia, heart failure, or even neurodegeneration (Yang et al., 2021), the findings hold much importance. Two proteins, namely, APOM, known to protect the lungs and kidneys from injuries (Ding et al., 2020), and APOA2, were also downregulated; similar observations were previously reported (Shen et al., 2020).

Functional enrichment analysis of the 38 differentially expressed proteins in severe versus non-severe cohort revealed that these proteins are enriched in pathways related to blood coagulation, fibrin clot formation, complement system, leukocyte activation, regulation of peptidase activity, regulated exocytosis, and extracellular structure organization, among others. Proteins like A2M, SERPINA4, SERPINA3, SERPING1, and FGG involved in regulated exocytosis of platelets were upregulated in the severe cohort suggesting an increased consumption of platelets. This could be a possible reason for the lower platelet count (clinically called thrombocytopenia) commonly reported in many severe cases of COVID-19 (Lippi et al., 2020), which is also associated with coagulation abnormalities, disease severity, and mortality (Bao et al., 2020; Liu et al., 2020; Yang et al., 2020). There is enough evidence to suggest that platelets have potent immune and inflammatory effector functions, in addition to their role in hemostasis. Interaction between viruses and platelets has been known to stimulate platelet degranulation leading to the release of a variety of cytokines and chemokines (Assinger, 2014; Seyoum et al., 2018). They also directly interact with leukocytes and endothelial cells to trigger and modulate inflammatory reactions and immune responses (Assinger, 2014). Thus, platelet hyperactivity due to the upregulation of these proteins correlates with the over-exuberant host inflammatory response as COVID-19 progresses from non-severe to severe.

Many peptidase activity regulator proteins, including SERPINA4, SERPING1, SERPINA3, SERPIND1, and A2M, are involved in blood coagulation and inflammation pathways. SERPINA4 is an inhibitor of the kallikrein–kinin system involved in coagulation and inflammation (Bryant and Shariat-Madar, 2009; Julie et al., 2016). SERPING1 is an inhibitor of the classical pathway of the complement system as well as of several proteins involved in blood coagulation (Kajdácsi et al., 2020). SERPIN A3 is a significant inhibitor of cathepsin G, a key proteolytic enzyme and inflammatory effector released by neutrophils (Kalsheker et al., 2006). A2M is an inhibitor of various proteases involved in blood coagulation and inflammation, including thrombin, kallikrein, plasmin, and cathepsin G (de Boer et al., 1993); SERPIND1 regulates blood clot formation by inhibiting thrombin (Salem and Thompson, 1987).

Moreover, the fibrinogen gamma chain or FGG is a component of the clotting factor fibrinogen, promoting tissue repair. High fibrinogen levels are associated with bleeding and thrombosis and correlate with the increased erythrocyte sedimentation rate (ESR) observed in severe cases (Eastham, 1954; Ghahramani et al., 2020). COVID-19 associated coagulopathy is common in severe patients (Connors and Levy, 2020), whereas overt disseminated intravascular coagulopathy, a critical condition characterized by abnormal blood clotting and bleeding, is observed in most critically ill patients who do not survive (Al-Samkari et al., 2020; Tang et al., 2020). These thrombotic complications can be characterized by dysregulation of proteins involved in blood coagulation, fibrin clot formation, and platelet exocytosis. Conversely, these proteins can be associated with disease severity and mortality risk and can serve as biomarkers for a better prognosis.

Consistent with previous studies, multiple acute phase proteins (APPs) like APCS, C4B, A2M, SERPING1, SERPINA3, and FGG were upregulated in severe patients (Li and Chen, 2020; Shen et al., 2020). APPs are manifested as the host innate response to any stress. Tissue damage caused by injury or infection instigates a local inflammatory response that leads to the release of pro-inflammatory cytokines. APPs are synthesized and released mainly by liver hepatocytes in response to these cytokines (Gruys et al., 2006). Severe COVID-19 patients tend to have higher pro-inflammatory cytokines (Blanco-Melo et al., 2020; Chen G. et al., 2020; Xiong et al., 2020), which explains the elevated APP levels and the acute inflammatory state correlating with disease severity. The complement system is a significant contributor to the acute phase response against infection. C4B is a proteolytic product of complement factor C4 and is involved in propagating all the three complement pathways (Mortensen et al., 2015) APCS or serum amyloid P component (SAP) is an activator of the classical pathway of the complement system (Yuste et al., 2007). Other proteins such as CFI, C8A, CFD, and CFP involved with the complement system were found to be downregulated in the severe cohort. Complement factor I (CFI) downregulates the complement system by inhibiting complement C3b and C4b (Roversi et al., 2011), while complement factor D (CFD) and properdin (CFP) play an essential role in the initiation and propagation of the alternate pathway in complement activation (Xu et al., 2001; Kouser et al., 2013). Complement activation is the first line of defense against invading pathogens. However, unrestrained and prolonged complement activation can lead to fatal consequences associated with severe COVID-19 cases (Gao T. et al., 2020; Holter et al., 2020; Noris et al., 2020). This disrupted fine-tune between the regulatory complement proteins is consistent with the prolonged systemic complement activation observed in severe patients.

Prediction of the outcome of the severity of COVID-19 has received massive attention worldwide in the healthcare environment. COVID-19 prognosis being a precipitous challenge, there is a need for protein biomarkers of severity that can estimate the level of severity to facilitate supplementary action at an earlier state. Here, we used the differentially expressed proteome between severe and non-severe COVID-19 for training a model to predict patients’ outcomes based solely on their proteome. SVM-based supervised learning approach has been used to process high dimensional data from the smaller data set. The SVM model could classify the predictors of severity by assigning the most probable marker in a descending order APCS, LCP1, SERPINA4, SEMG2, LPA, C4B, PI16, SERPINA7, S100A8, SERPINA3, SERPINA6, FGG, TPM4, IGFBP3, CA1, SERPIND1, CFD, AZGA, GPX3, and APOB a Variable Importance in Projection Score. The protein biomarkers of severity can be translated by creating panels that can help clinicians segregate patients based on the biomarker features with potential to determine the severity of the disease.

Multiple antiviral drug therapies and clinical drug trials are ongoing to develop a definitive solution for this life-threatening viral infection. Current approaches of treating various stages of COVID-19 patients with commercially available drugs include two major categories: treatment with antiviral drugs and immune modulators. HIV protease inhibitors are quite famous as they belong to the former category, but no definitive studies have proven those drugs to be potent inhibitors, driving the quest for an effective treatment (Shah et al., 2020). Our study performed *in silico* drug repurposing analysis with 9 proteins from our proteomic analysis against a library of 58 small molecules. The chosen drugs are previously found to target the protein–protein interactions that occur between SARS-CoV-2 and human proteins in a cell line model (Gordon et al., 2020). Two FDA-approved drugs, selinexor and ponatinib, were found to inhibit most of the proteins belonging to two different cohorts: COVID-19 positive vs. COVID-19 negative and non-severe vs. severe. The FDA has approved selinexor to treat multiple myeloma combined with dexamethasone (Chari et al., 2019). The drug is a first-class exportin-1 (XPO1) inhibitor that brings apoptosis in cancer cells by blocking nucleocytoplasmic transport of tumor suppressor proteins (Richter et al., 2020). Although developed originally as anticancer drugs, exportin inhibitors can act as antiviral drugs as they have the potential to block the intracellular replication of viral particles by inhibiting the transport of viral replication proteins into the cytoplasm (Uddin et al., 2020). Hence, the drug is currently under phase II clinical trial for COVID-19 infection (ClinicalTrials.gov Identifier: NCT04349098). Five plasma proteins from our study, including thyroxine-binding globulin (SERPINA7) and heparin cofactor 2 (SERPIND1) belonging to the family of serine protease inhibitors (SERPINs), were shown to interact with selinexor, suggesting these SERPINs could be a target for the drug. Both proteins are also seen to interact with another FDA-approved drug called ponatinib. Originally, the tyrosine kinase inhibitor ponatinib was used to treat chronic myeloid leukemia (Tan et al., 2019). It is currently not under any clinical trial for treatment of COVID-19 patients, but recent studies in mice models showed it could suppress the cytokine storms from viral infections like influenza (Chen et al., 2019). Hence, ponatinib, as an immune modulator, appears to be a suitable drug for making therapeutic cocktails against COVID-19 infection in the future. The potential drug candidates identified by *in silico* docking should be validated using *in vitro* cell line model.

## Conclusion

The comprehensive proteomics study of COVID-19 infected patient plasma emphasizes that the mass spectrometry–detected host proteins hold the potential for monitoring the disease severity progression and the drug targets identified might aid in therapeutic interventions. Several proteins such as AGT, FGG, APOB, SERPING1, and SERPINA3 identified using quantitative proteomics techniques were further validated using a targeted mass-spectrometry approach. Using supervised machine learning–based approach, the proteins such as APCS, LCP1, SERPINA4, SEMG2, LPA, C4B, PI16, SERPINA7, S100A8, SERPINA3, SERPINA6, FGG, TPM4, IGFBP3, CA1, SERPIND1, CFD, AZGA, GPX3, and APOB were identified to be the most probable classifiers of disease severity. However, considering the limited sample size, the predicted panel of proteins should be further validated in a large cohort of the patient samples for successful clinical translation. The *in silico* docking studies identified two potential FDA-approved drugs, namely, selinexor and ponatinib, binding to the proteins SERPIND1, SERPINA7, and S100A9 involved in the pathway related to regulation of peptidase activity. Thus, the present study reveals a set of potential prognosis markers and drug candidates for circumventing the COVID-19 infection.

## Code Availability

The custom code for machine learning has been deposited at https://github.com/GaurishLoya/Machine_learning_prediction_Model.

## Data Availability Statement

All proteomics data associated with this study are present in the manuscript or the Supplementary Material. Raw MS data and search output files for proteomics datasets are deposited to the ProteomeXchange Consortium via the PRIDE partner repository with the dataset identifier PXD022296 (Link: http://www.ebi.ac.uk/pride). Targeted proteomic data is deposited on Panorama Public, and the ProteomeXchange ID reserved for the data is PXD022475. It can be accessed using the access URL-https://panoramaweb.org/COVID_PLASMA.url.

## Ethics Statement

The studies involving human participants were reviewed and approved by Institutional Review Board Kasturba Hospital for Infectious Diseases, Mumbai and Institute Ethics Committee, Indian Institute of Technology Bombay, Mumbai. Written informed consent for participation was not required as the samples used in this study were leftover samples from routine hematological tests.

## Author Contributions

SS, JS, and OS conceptualized and designed the study. SA, RB, VP, and AkS collected the samples for the study. KS, MC, SuG, RB, and ArB prepared the samples. ArB, SaG, and SS optimized the mass spectrometry method and final runs on the instrument. MP, SaG, SS, JR, and AlS carried out the MRM experiments and data analysis using Skyline. DB, AvS, AV, AA, KS, and SS performed the statistical analysis and data visualization. DB and GL performed prediction analysis using machine learning. AnB, AV, AM, and KM performed the docking study. SS, KS, AA, MC, ApB, SuG, SB, RB, DB, AV, AlS, and AM wrote and reviewed the manuscript. All authors contributed to the article and approved the submitted version.

## Conflict of Interest

The authors have filed an Indian patent related to this work “Protein markers and method for prognosis of COVID-19 in individuals” (Application number: 202023054753). The authors declare that the research was conducted in the absence of any commercial or financial relationships that could be construed as a potential conflict of interest.

## Footnotes

1 https://in.mathworks.com/help/stats/classification.html

2 https://scikit-learn.org/stable/index.html

3 https://pyrx.sourceforge.io/

## Supplementary Material

Supplementary Figure 1 Figure representing the correlation matrix of total 74 plasma sample of COVID-19 patients including 20 negative patients, 18 non-severe patients, and 36 severe patients.

Supplementary Figure 2 The figure shows two chromatograms TIC and base peak chromatogram. The TIC chromatogram shows all ions from each mass spectrometer scan (a given time period) summed and plotted as a function of time. The base peak chromatogram shows all ions from the base peak (the most intense mass peak in the mass spectrum) summed and plotted as a function of time. Mass spectrometry instrument settings used for analysis of COVID-19 positive and negative plasma samples are given below the figure.

Supplementary Figure 3 Principal component analysis of COVID-19 severe and COVID-19 non-severe patients. **(A)** The variance over PC index plot. **(B)** Depicts the PCA clustering of the severe versus non-severe patients in 2D and 3D.

Supplementary Figure 4 Proteomic analysis of COVID-19 severe and COVID-19 negative patients. **(A)** Top 25 differentially expressed proteins of severe and negative in the form of heatmap. **(B)** Depicts the PLSDA clustering of the severe vs. negative patients.

Supplementary Figure 5 ROC–AUC curve of SVM model. **(A)** The ROC–AUC curves of targets in the non-severe class. **(B)** Depicts the ROC–AUC curves of targets in the severe class.

Supplementary Figure 6 BSA as a QC standard to monitor day-wise instrument response. **(A)** Gives an overview of all the peptides of BSA that were monitored and their approximate retention times. The peptides of BSA monitored were DLGEEHFK, LVNELTEFAK, DDSPDLPK, AEFVEVTK, HLVDEPQNLIK, LGEYGFQNALIVR, and QTALVELLK. **(B)** MRM peaks for LVNELTEFAK, DDSPDLPK, and HLVDEPQNLIK, respectively. **(C)** The MRM peak shapes of QTALVELLK for five consecutive days. **(D,E)** Peak area and retention time of QTALVELLK for the same 5 days. Note the consistency in peak shapes, peak areas, and retention times representing the consistency in the instrument’s response.

Supplementary Figure 7 Spiked-in synthetic peptide as QC standard to check for variability in between sample runs, if any. The peptide used was FEDGVLDPDYPR with a heavy labeled arginine. **(A–C)** The MRM peak shapes, peak areas, and retention time, respectively, for six randomly picked samples from our MRM runs.

Supplementary Figure 8 **(A–I)** The *in silico* molecular docking of target proteins with potential drug candidates and their respective control inhibitor. These drugs have equal or higher binding affinity than the control drug (i); also, they share the same binding pocket with the respective control inhibitors. The potential FDA-approved drugs identified to target are also shown in the figure (ii). The structures of the drugs are obtained from the ZINC15 database.

Supplementary Table 1 Demographic characteristics of COVID-19 patients.

Supplementary Table 2 Clinical information of COVID-19 patients.

Supplementary Table 3 List of DEPs altered in COVID-19 positive vs. negative.

Supplementary Table 4 List of DEPs altered in COVID-19 severe vs. non-severe.

Supplementary Table 5 Consolidated sheet of machine learning output file.

Supplementary Table 6 List of peptides and transitions used for MRM analysis.

Supplementary Table 7 List of proteins involved in the enriched pathways.

Supplementary Table 8 List of FDA approved, Clinical and Pre-clinical drugs used in docking study.

Supplementary Table 9 The potential drug binding to proteins up-regulated in COVID-19 severe.

Supplementary Table 10 The list of potential drug binding to the target proteins.

Supplementary Table 11 The list of proteins selected for docking.

Supplementary Table 12 The potential drugs binding to proteins upregulated in COVID-19 positive.
