# Supplementary material for: Proteomics and Machine Learning Approaches Reveal a Set of Prognostic Markers for COVID-19 Severity With Drug Repurposing Potential
Source: Front Physiol. 2021 Apr 27;12:652799. doi: 10.3389/fphys.2021.652799 (PMC8120435; doi:10.3389/fphys.2021.652799)
Supplement: Supplementary Figure 1 — Figure representing the correlation matrix of total 74 plasma sample of COVID-19 patients including 20 negative patients, 18 non-severe patients, and 36 severe patients. [file Data_Sheet_1.docx]

**Supplementary Data sheet**

**Standardization of plasma sample collection and processing**

Standardized blood collection and pre-analytical procedures should be performed. This work was carried out during extreme lockdown time in Mumbai. Hence, several lab members were working simultaneously at Kasturba hospital and IIT Bombay for immediate and careful processing of samples. The blood collection task was further divided into three steps namely, collection of blood from patients, separation of plasma and heat inactivation of the samples. One person in the hospital was allotted for careful monitoring of time and storage of the sample. The sample processing task was split into the following task of optimization of the method, plasma depletion, digestion and desalting. Care was taken that each task was performed by a single person and was consistent throughout. The optimization of plasma samples for MS analysis was done using previously available samples in the lab. The heat-inactivated plasma samples were transported in random batches to IIT Bombay from Kasturba Hospital, Mumbai. The samples were immediately processed for mass-spectrometry analysis and the clinical details were available only after processing. Although no randomization was performed, the sample processing was unbiased considering it was a blinded study.

Supplementary Figure-S1


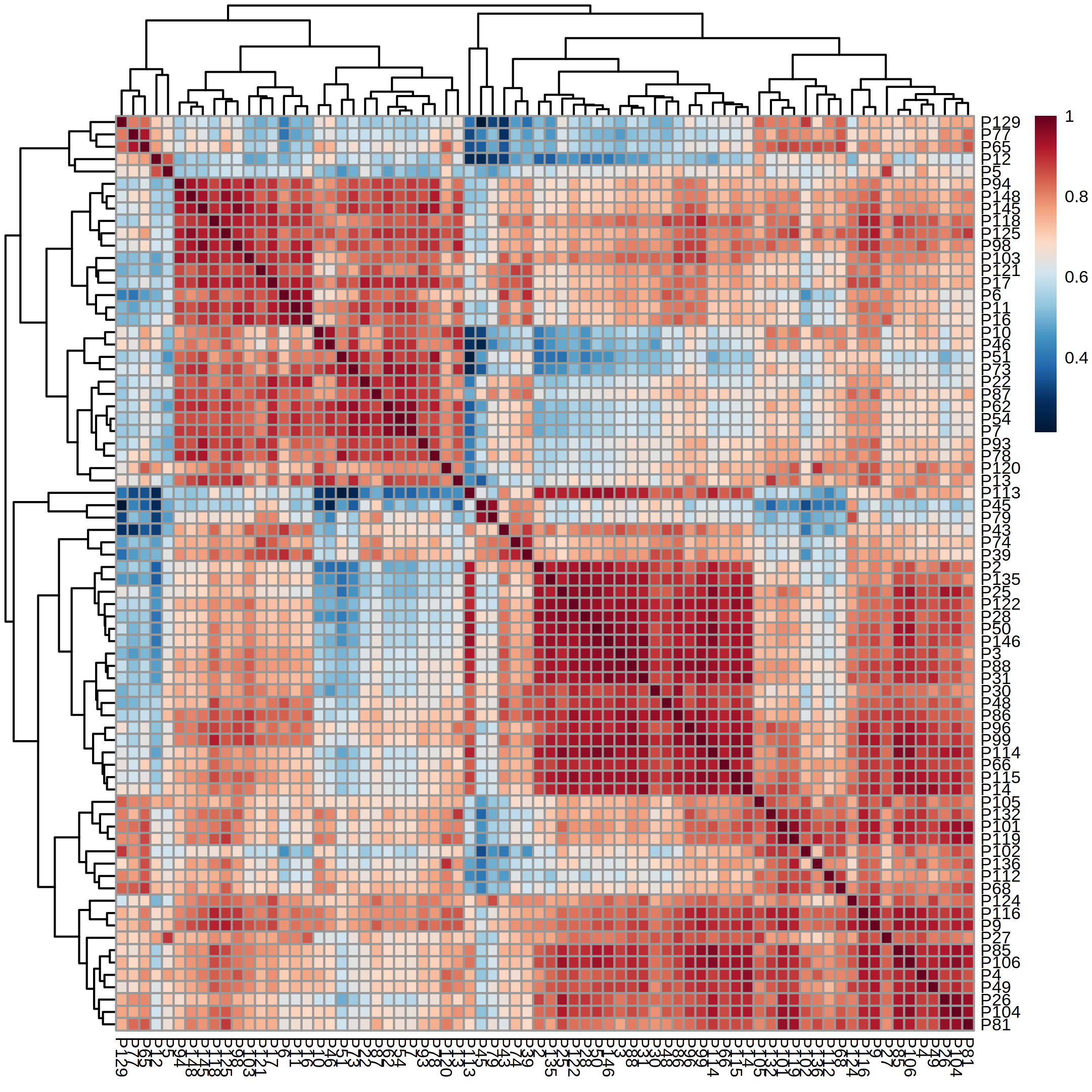


Figure-S1: Figure representing the Correlation matrix of total 74 plasma sample of COVID-19 patients including Negative- 20 patients, Non-Severe- 18 patients and Severe-36 patients


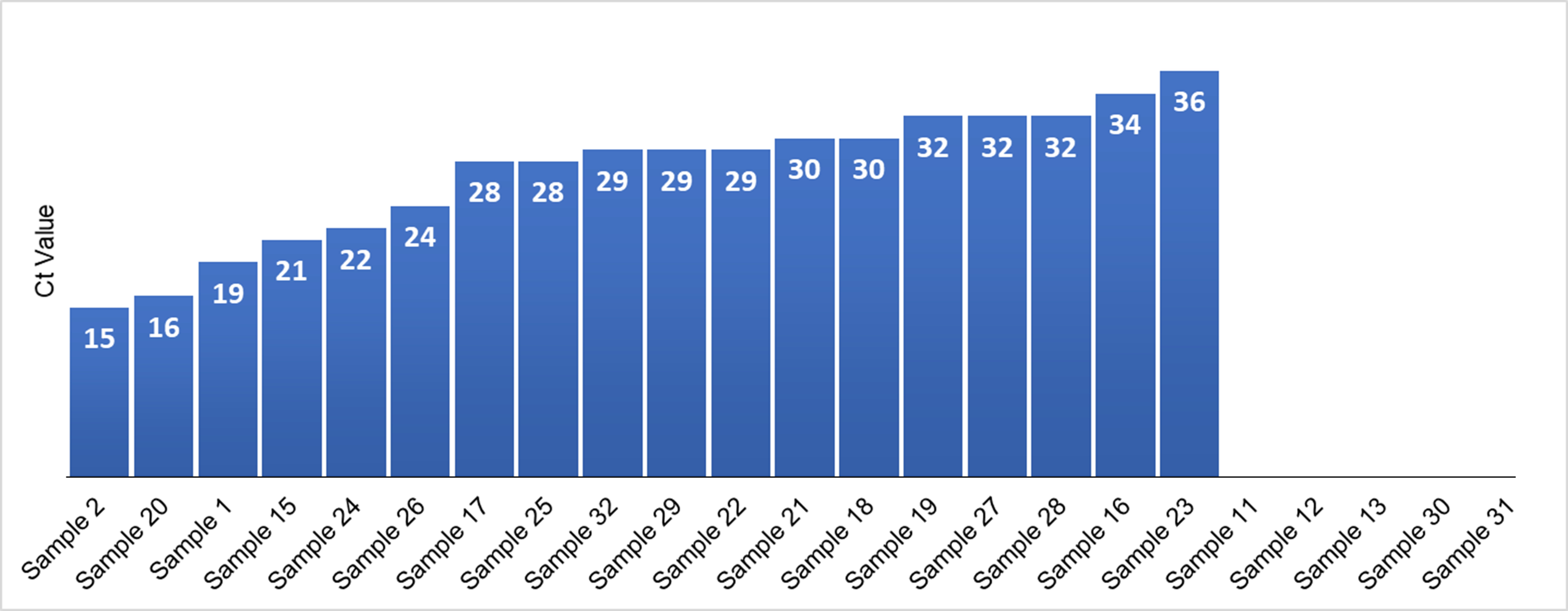
Supplementary Figure-S2


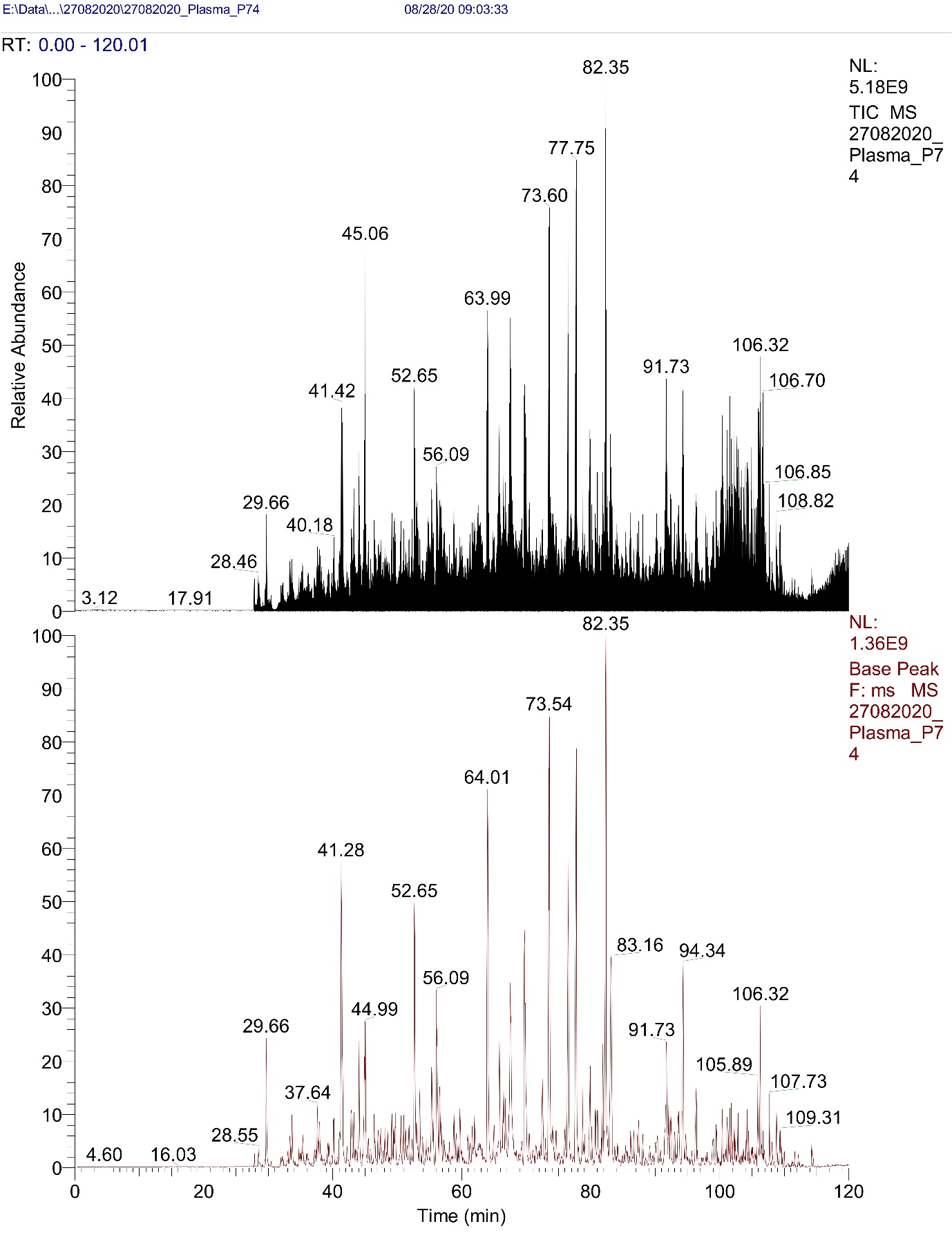


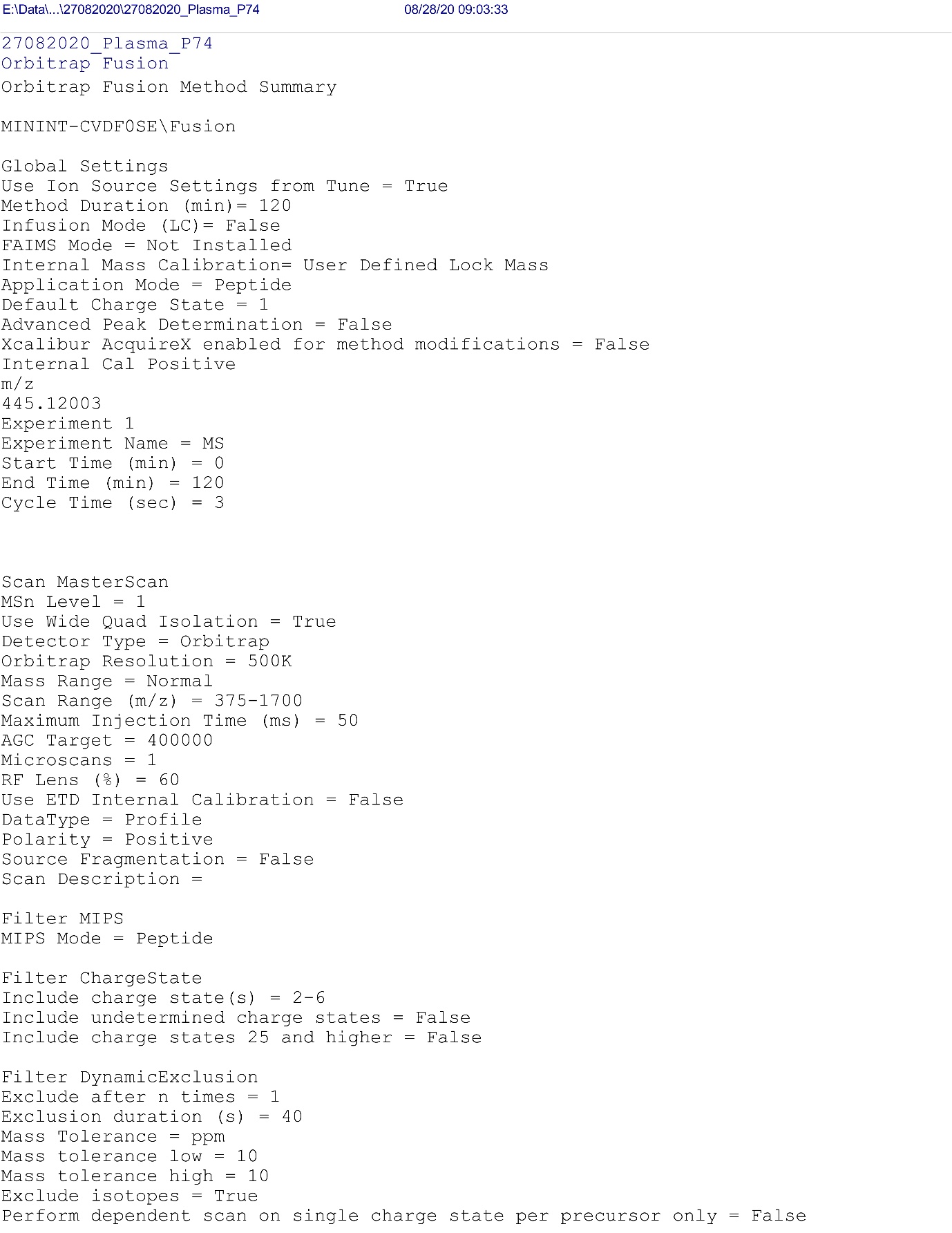

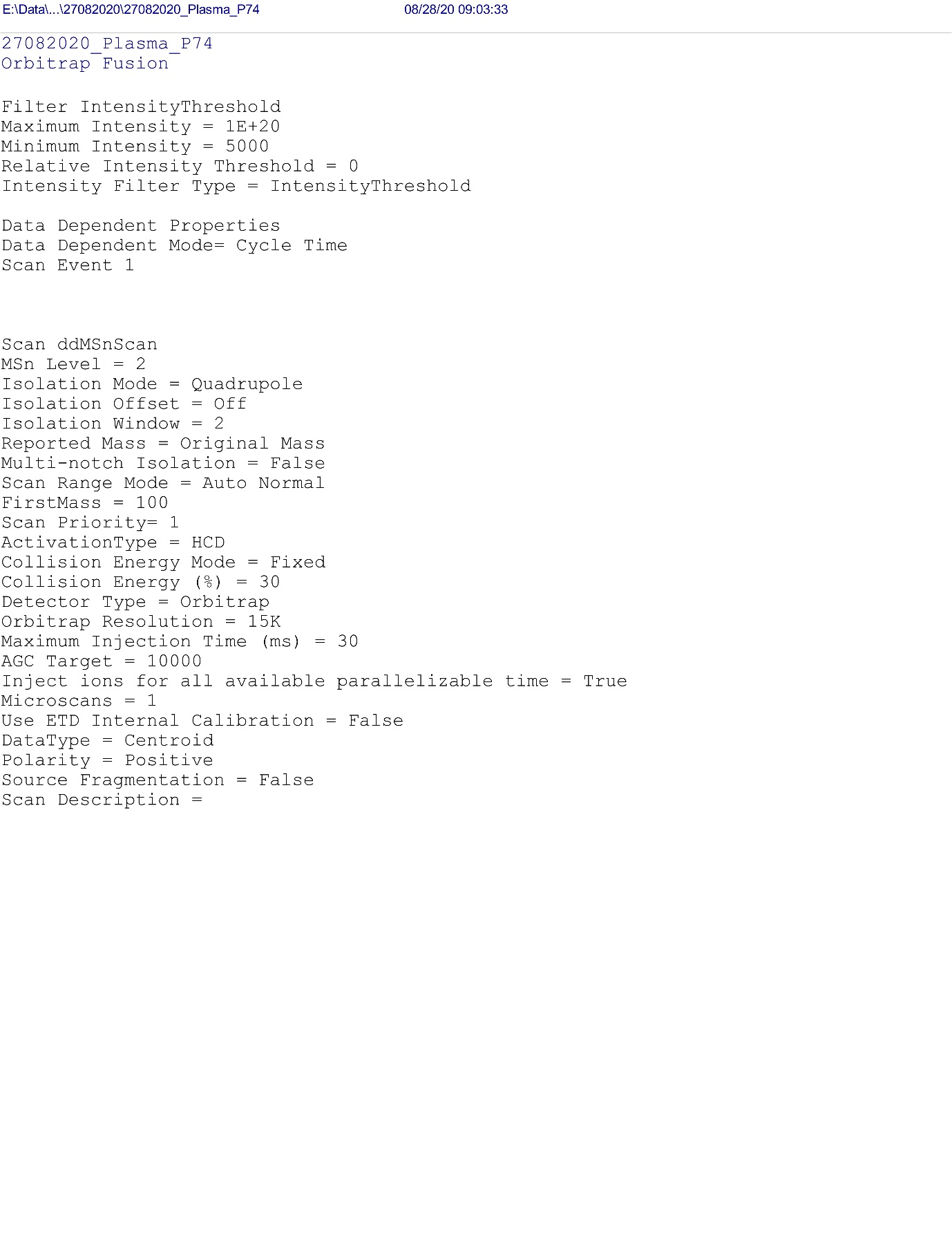


Figure-S2: The figure shows two chromatograms TIC and base peak chromatogram. The TIC chromatogram shows all ions from each mass spectrometer scan (a given time period) summed and plotted as a function of time. The base peak chromatogram shows all ions from the base peak (the most intense mass peak in the mass spectrum) summed and plotted as a function of time. Mass Spectrometry instrument setting used for analysis of COVID-19 Positive and Negative plasma sample are given below the figure.

Supplementary Figure-S3

**A**


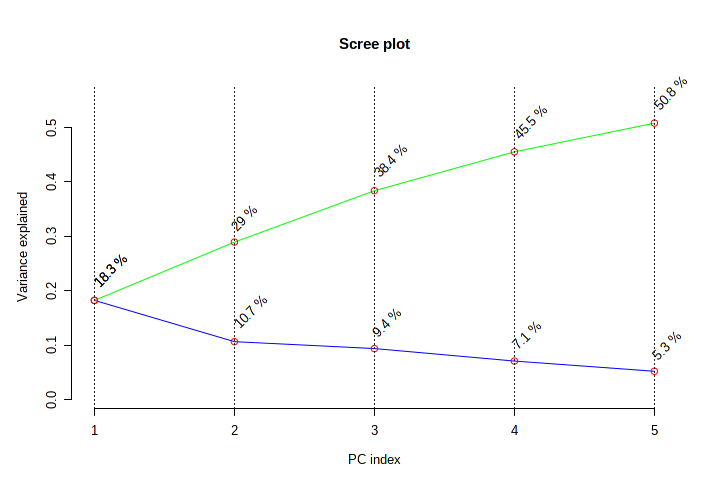


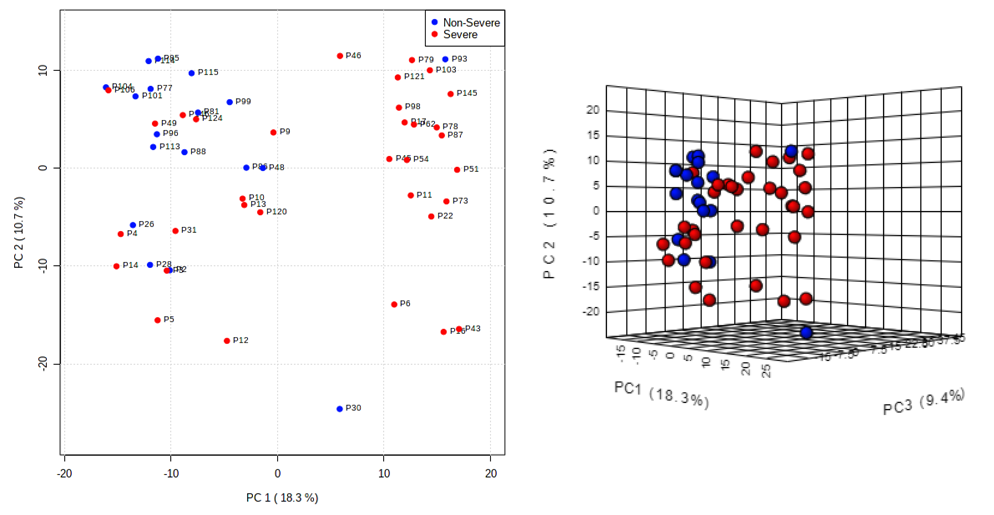


**B**

Figure-S3: Principal Component Analysis of COVID-19 Severe and COVID-19 Non-Severe patients. Figure A represent the variance over PC index plot; Figure B depicts the PCA clustering of the Severe versus Non-Severe patients in 2D and 3D.

Supplementary Figure-S4
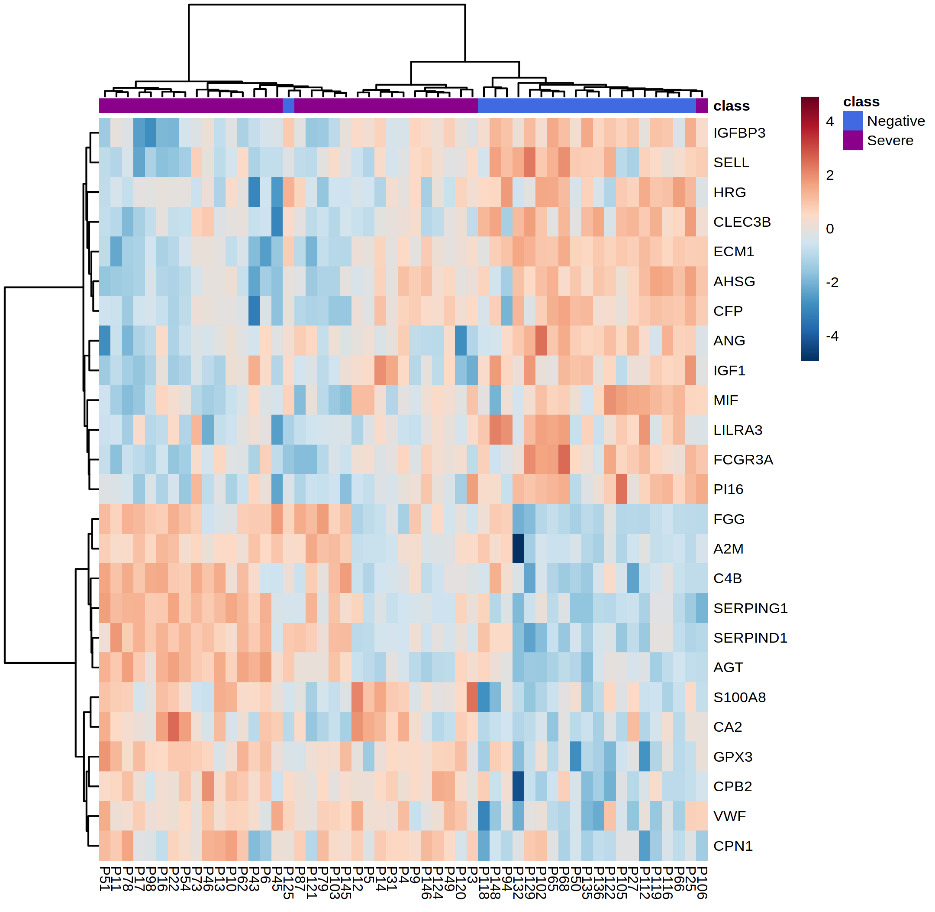


**B**

**A**


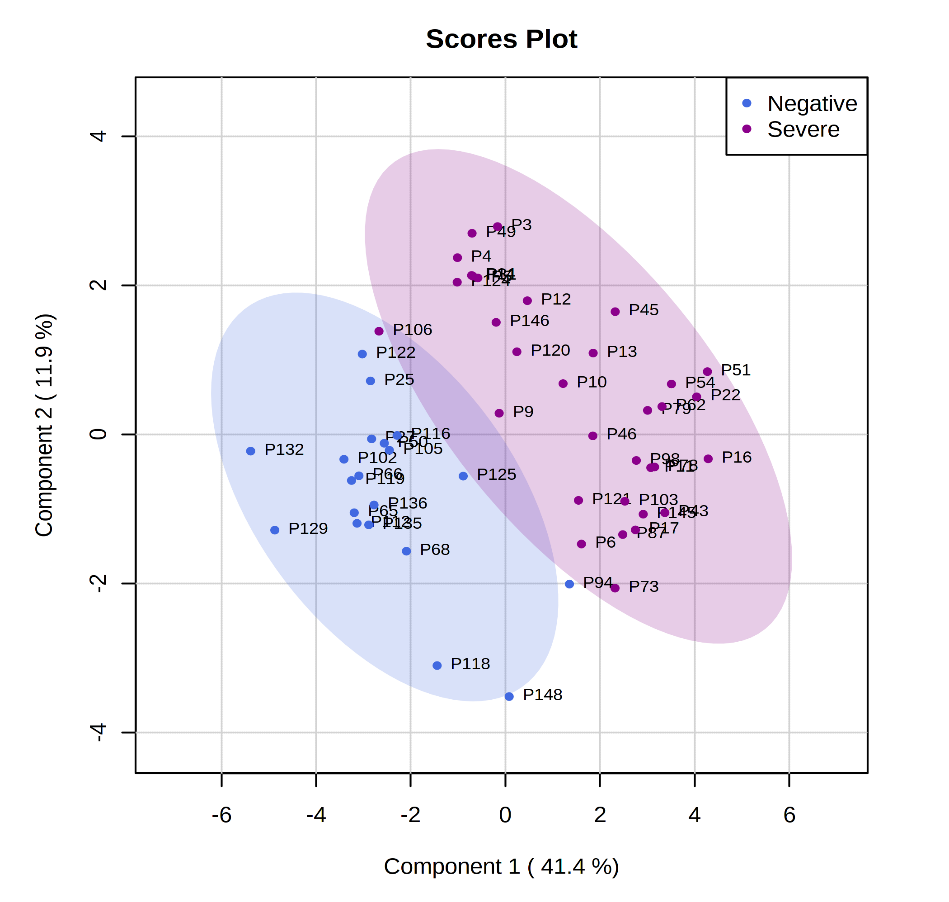


Figure-S4: Proteomic analysis of COVID-19 Severe and COVID-19 Negative patients. Figure A represent top 25 differentially expressed of Severe and Negative in the form of Heatmap; Figure B depicts the PLSDA clustering of the Severe versus Negative patients

Supplementary Figure-S5

**A**


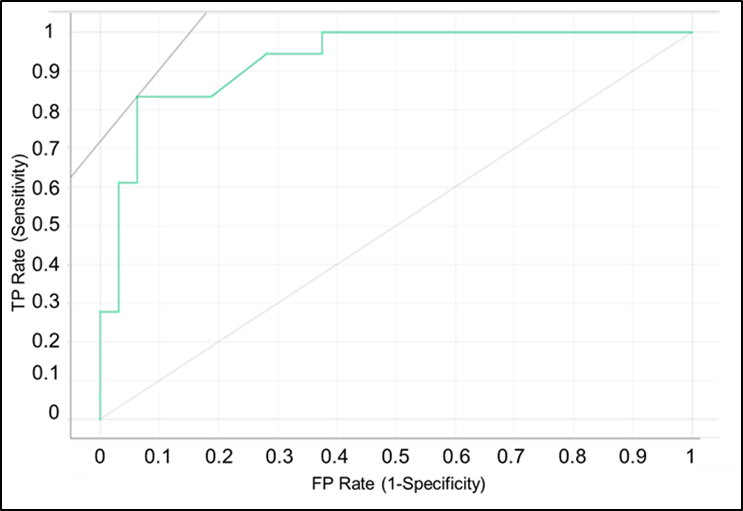


**B**


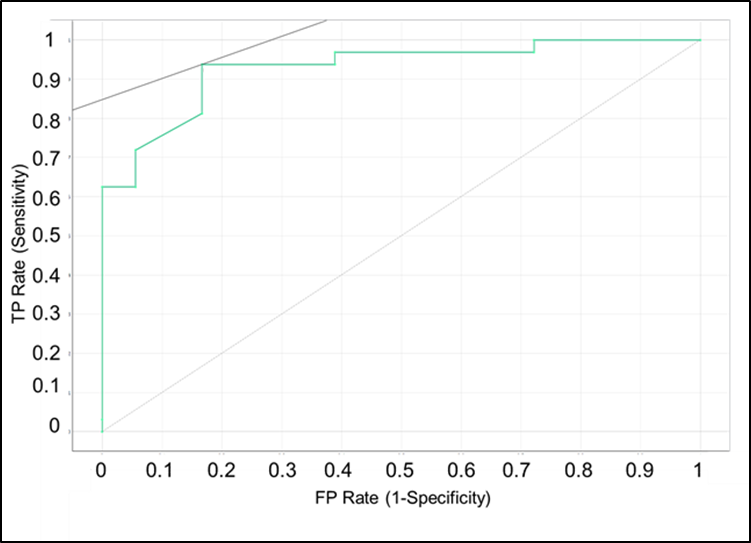


Figure S-5: ROC-AUC Curve of SVM model. Figure A represent the ROC-AUC curves of Target Non-Severe class; Figure B depicts the ROC-AUC curves of Target Severe class

Supplementary Figure-S6


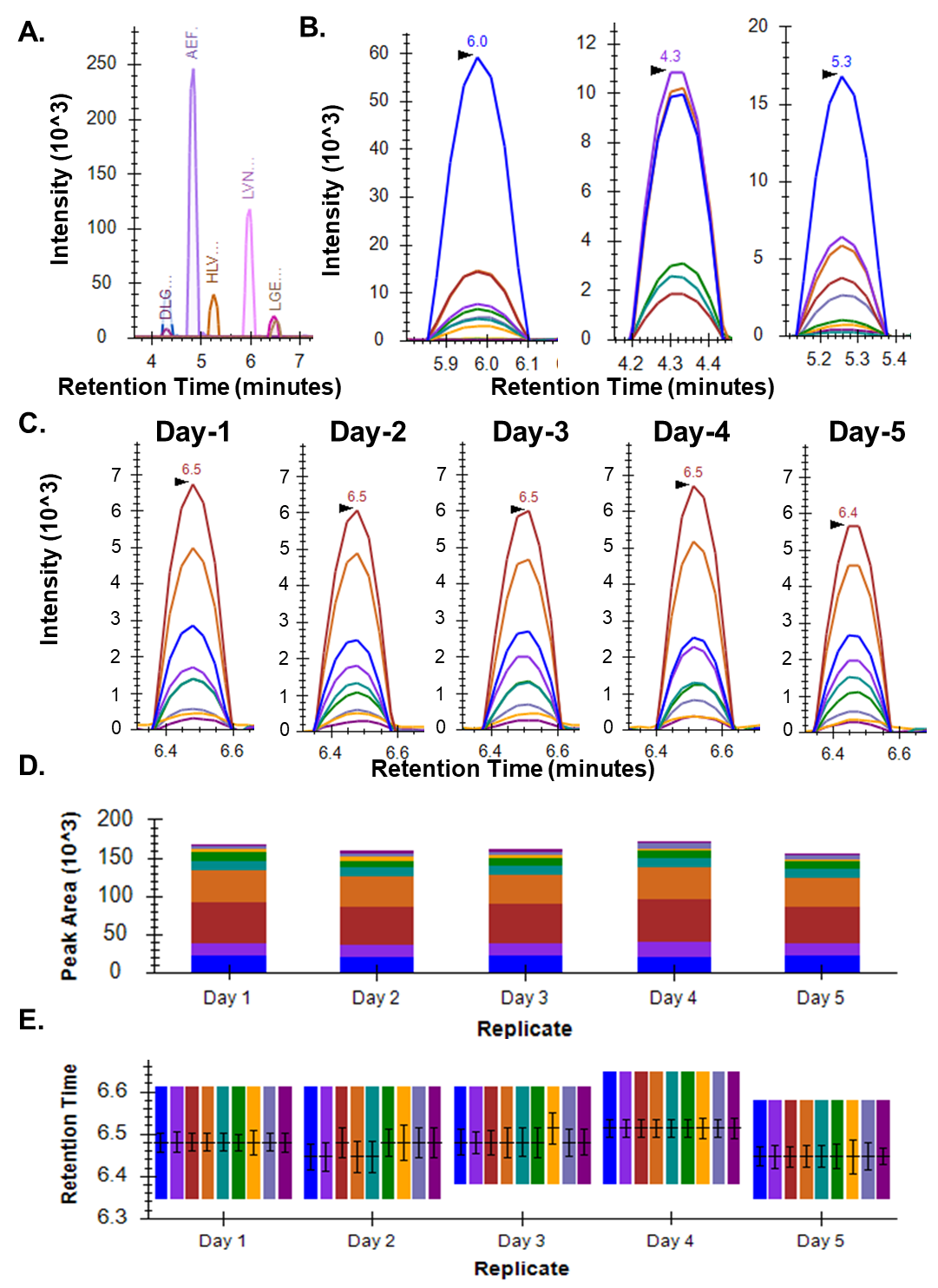


Figure-S6: BSA as a QC standard response to monitor day-wise instrument response Figure A gives an overview of all the peptides of BSA that were monitored and their approximate retention times. The peptides of BSA monitored were DLGEEHFK, LVNELTEFAK, DDSPDLPK, AEFVEVTK, HLVDEPQNLIK, LGEYGFQNALIVR and QTALVELLK. Figure B shows representative MRM peaks for LVNELTEFAK, DDSPDLPK and HLVDEPQNLIK, respectively. Figure C gives the MRM peak shapes of QTALVELLK for five consecutive days. Figure D and Figure E show peak areas and retention time of QTALVELLK for the same five days. Note the consistency in peak shapes, peak areas and retention times representing the consistency in the instrument’s response.

Supplementary Figure-S7


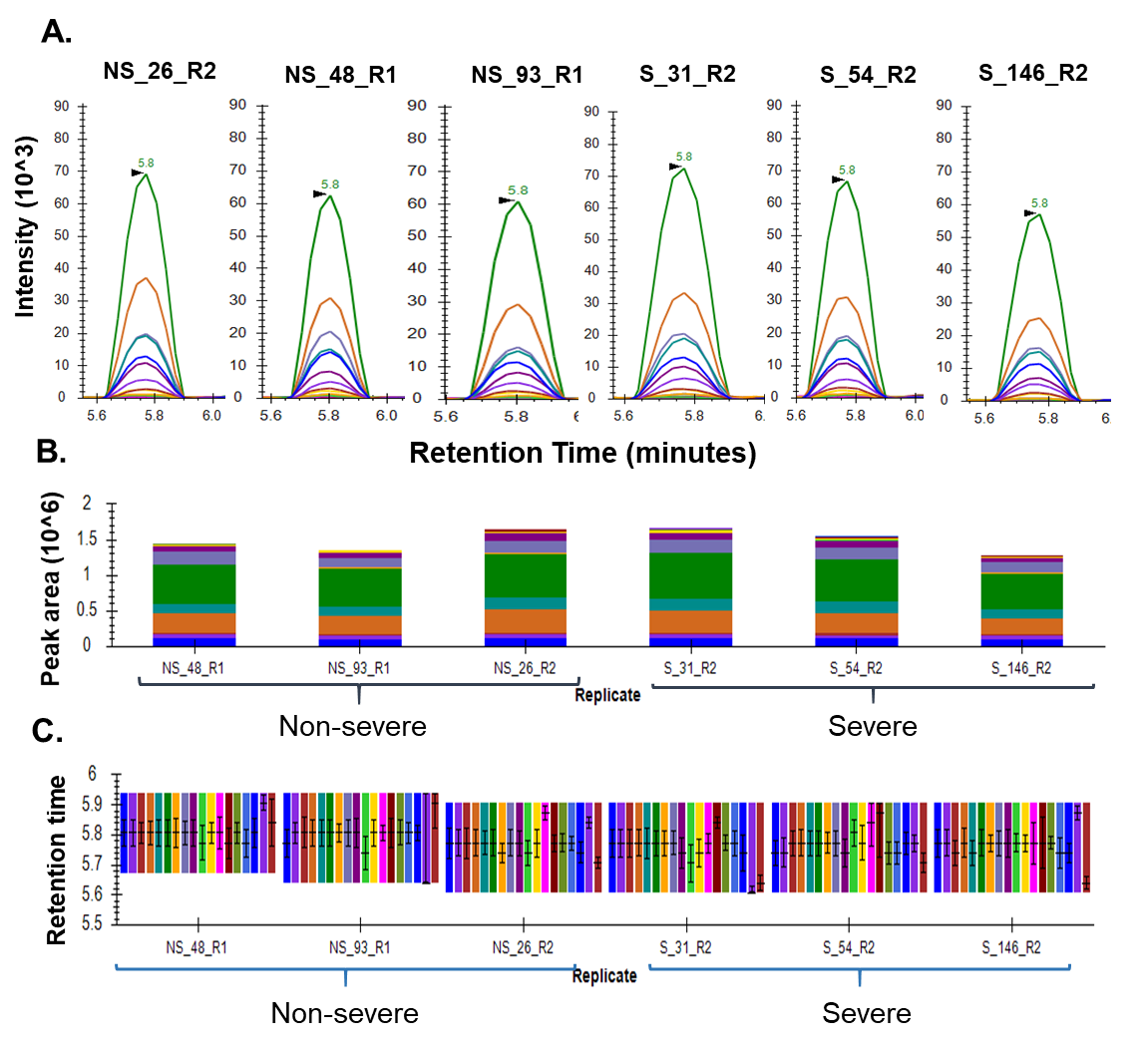


Figure-S7: Spiked in synthetic peptide as QC standard to check for variability in between sample run, if any. The peptide used was FEDGVLDPDYPR with a heavy labelled arginine. Figure A, B and C show the MRM peak shapes, peak areas and retention time, respectively for 6 randomly picked samples from our MRM runs.

Supplementary Figure- S8


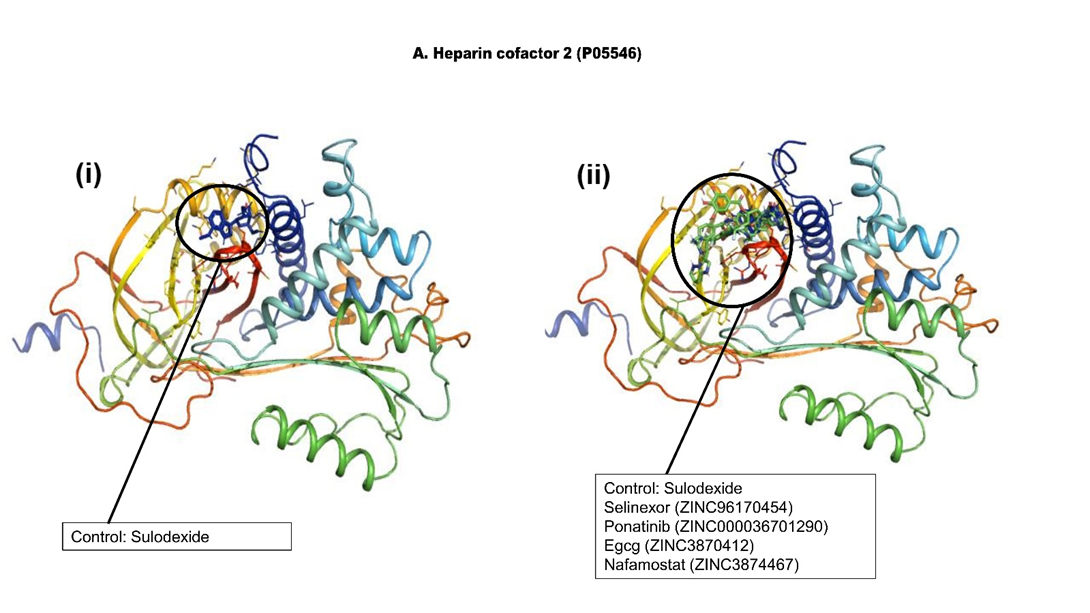


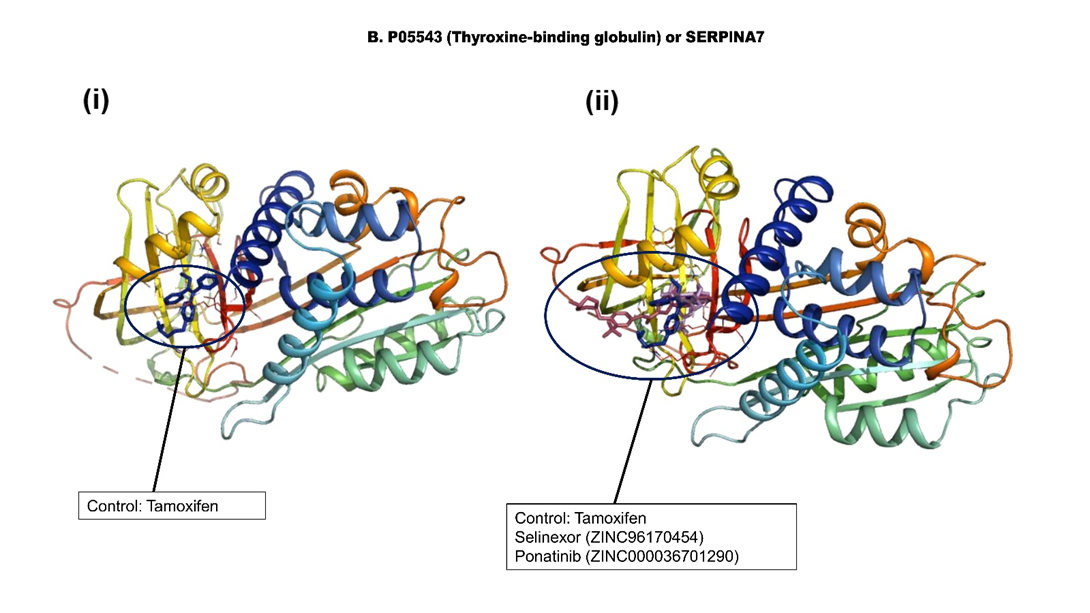


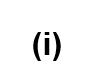

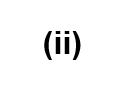

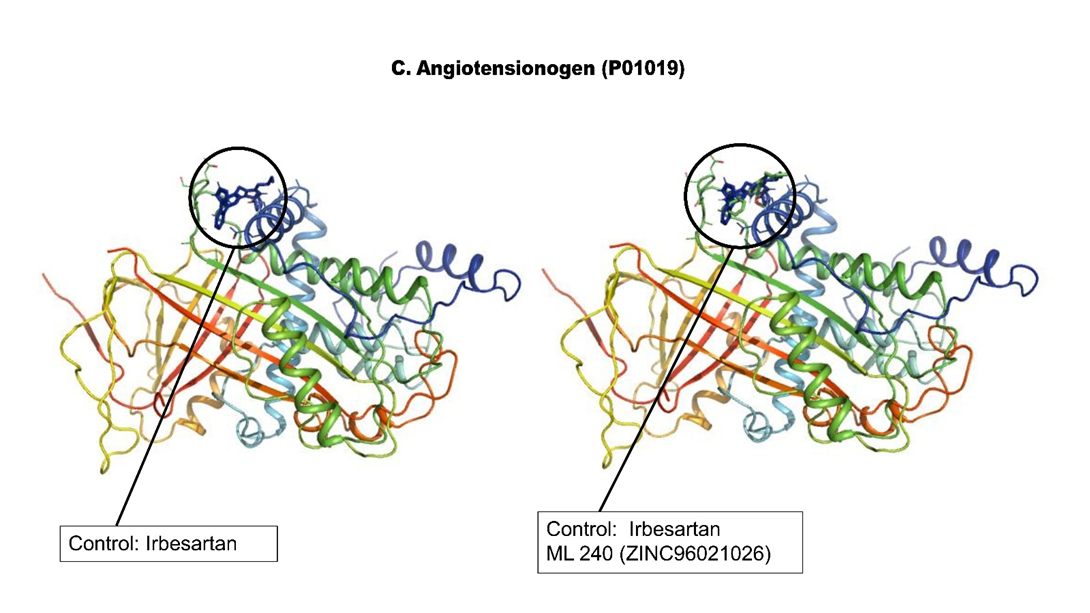


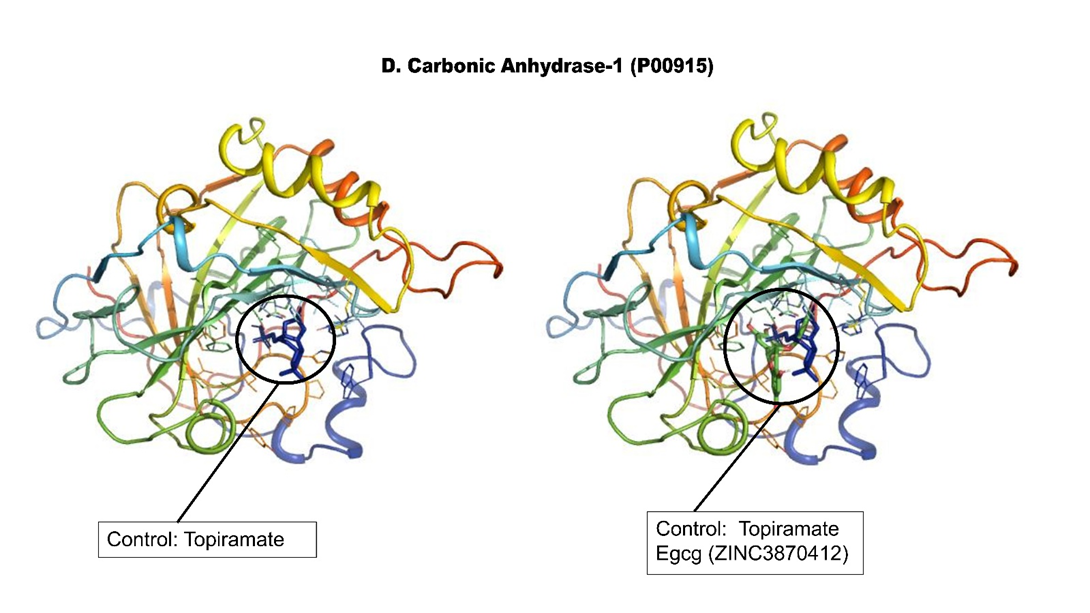


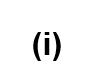

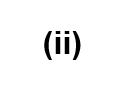


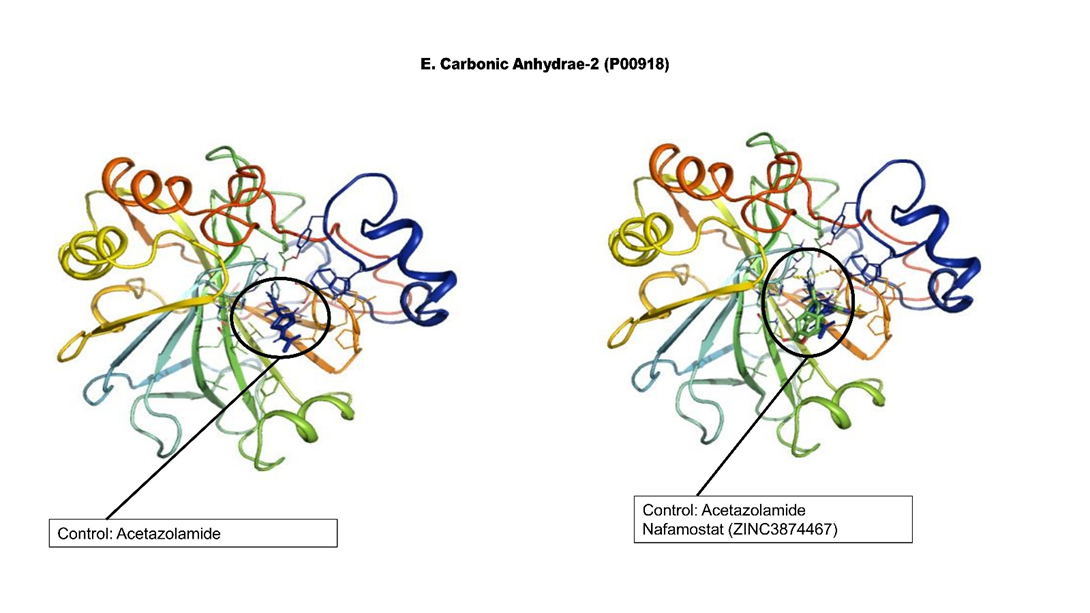


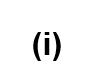

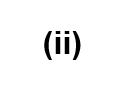


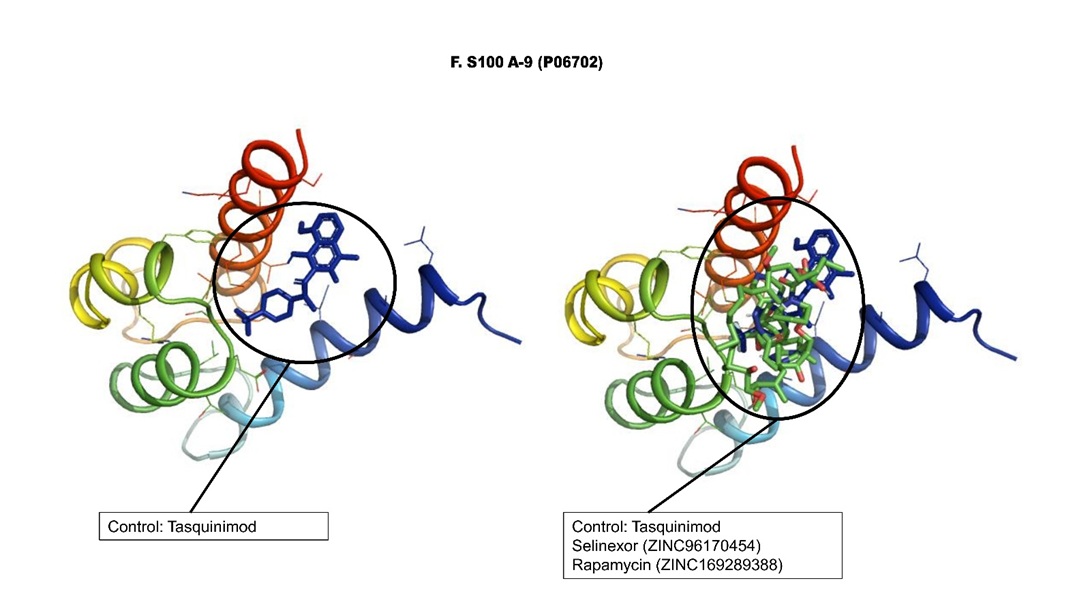


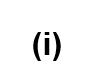

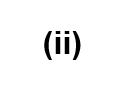


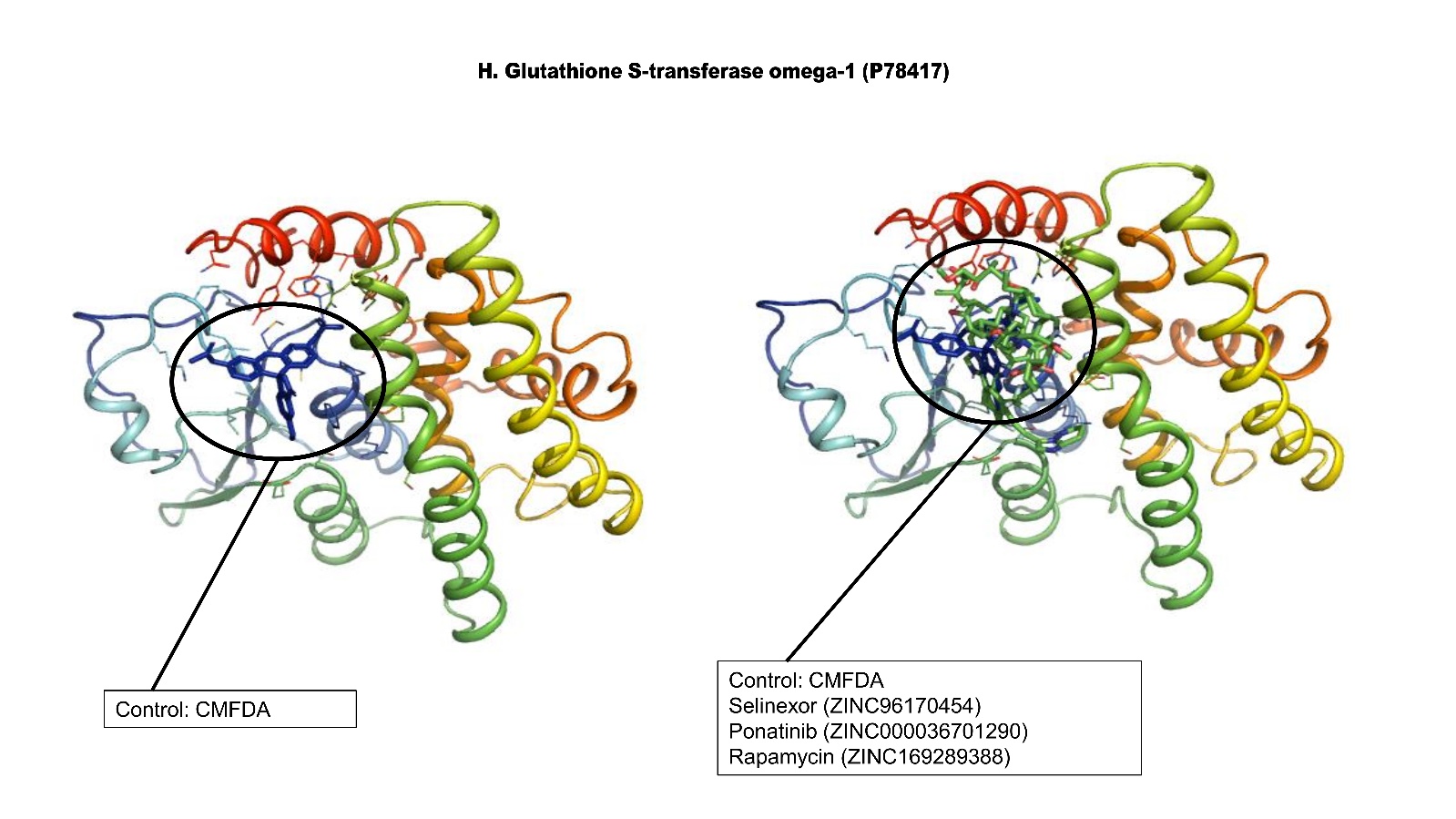

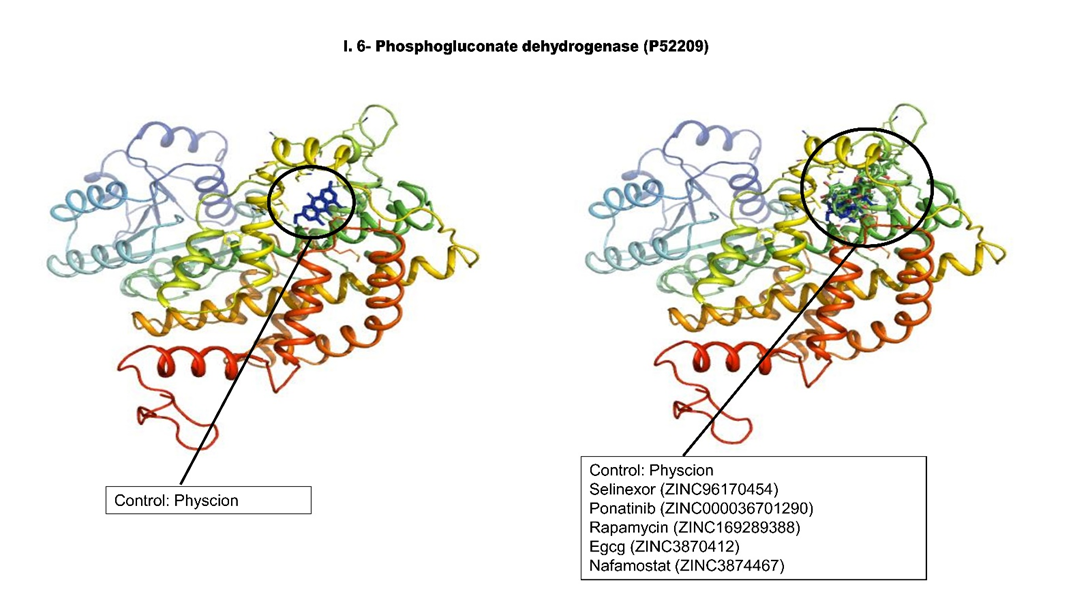

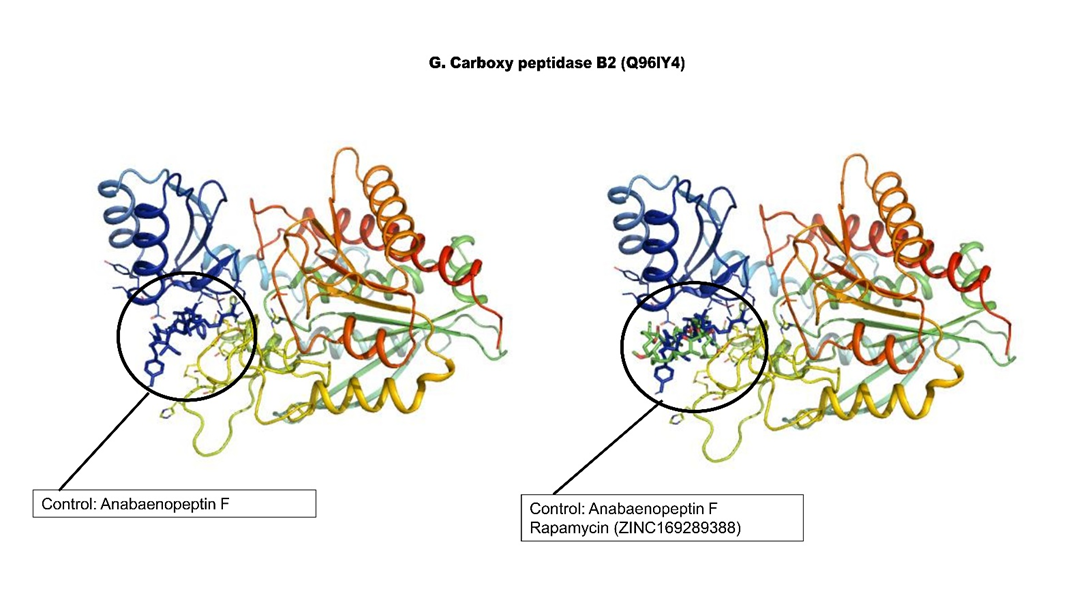


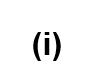

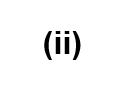

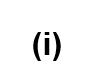

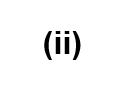

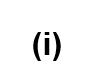

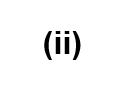


Figure-S8: Figure A to Figure I show the *In-silico* molecular docking of target proteins with potential drug candidates and their respective control inhibitor. These drugs have equal or higher binding affinity than the control drug (i); also, they share the same binding pocket with the respective control inhibitors. The potential FDA approved drugs identified to target are also shown in the figure (ii). The structures of the drugs are obtained from the ZINC15 database.

**Data availability**

All proteomics data associated with this study are present in the manuscript or the Supplementary Materials. Raw MS data and search output files for proteomics datasets are deposited to the ProteomeXchange Consortium via the PRIDE partner repository with the dataset identifier PXD022296 (Link: https://www.ebi.ac.uk/pride/archive/login, Username: reviewer_pxd022296@ebi.ac.uk, Password: Ewqtl0NJ). Targeted proteomic data is deposited on Panorama Public, and the ProteomeXchange ID reserved for the data is PXD022475. It can be accessed using the access URL-https://panoramaweb.org/COVID_PLASMA.url.
