# Supplementary material for: Proteomics and Machine Learning Approaches Reveal a Set of Prognostic Markers for COVID-19 Severity With Drug Repurposing Potential
Source: Front Physiol. 2021 Apr 27;12:652799. doi: 10.3389/fphys.2021.652799 (PMC8120435; doi:10.3389/fphys.2021.652799)
Supplement: Supplementary Table 1 — Demographic characteristics of COVID-19 patients. [file Table_1.docx]

**Supplementary Table-S1. Demographic characteristics of COVID-19 patients.**

|  | **Negative**  **(n = 20)** | **Non- Severe**  **(n = 18)** | **Severe**  **(n = 33)** |
| --- | --- | --- | --- |
| **Gender-no. (%)** |  |  |  |
| Males | **14 (70%)** | **11 (61.1%)** | **24 (72.7%)** |
| Females | **6 (30%)** | **7 (38.9%)** | **9 (27.7%)** |
| **Age (Median and range)** | **39(86-18)** | **59 (74-50)** | **56 (90-25)** |
| **Symptoms on admission (%)** |  |  |  |
| Fever | **75%** | **94.4%** | **72.7%** |
| Cough | **50%** | **78.9%** | **63.6%** |
| Breathlessness | **35%** | **61.1%** | **87.8%** |
| Sore throat | **20%** | **27.7%** | **12.1%** |
| General weakness | **35.65%** | **44.4%** | **42.4 %** |
| Respiratory illness | **None** | **5.55%** | **81.8%** |
| **Days of hospitalization (Median and range)** | **4 (9 - 1)** | **10 (15 - 3)** | **17 (96 - 1)** |
| **Comorbidities (%)** |  |  |  |
| Diabetes | **None** | **44.4%** | **66.6%** |
| Hypertension | **5%** | **33.3%** | **60.6%** |
| **Treatment (%)** |  |  |  |
| Immunomodulatory | **10%** | **27.7%** | **66.6%** |
| Antiviral | **5%** | **22.2%** | **84.8%** |
| Antibiotics | **55%** | **55.5%** | **93.9%** |
| Oxygen inhalation | **None** | **27.7%** | **96.9%** |
| **Patient outcomes (%)** |  |  |  |
| Discharged | **100%** | **100%** | **54.6%** |
| Dead | **None** | **None** | **45.4%** |
