# Supplementary material for: Proteomics and Machine Learning Approaches Reveal a Set of Prognostic Markers for COVID-19 Severity With Drug Repurposing Potential
Source: Front Physiol. 2021 Apr 27;12:652799. doi: 10.3389/fphys.2021.652799 (PMC8120435; doi:10.3389/fphys.2021.652799)
Supplement: Supplementary Table 3 — List of DEPs altered in COVID-19 positive vs. negative. [file Table_3.docx]

**Supplementary Table-S3.**

**List of differentially expressed proteins in the COVID-19 Positive versus COVID-19 Negative plasma sample**

| **S.No.** | **Protein IDs** | **Protein Name** | **Gene** | **Fold Change** | **Trend** |
| --- | --- | --- | --- | --- | --- |
| 1 | P13645 | Keratin, type I cytoskeletal 10 | KRT10 | 3.20109 | Up |
| 2 | P35908 | Keratin, type II cytoskeletal 2 epidermal | KRT2 | 2.89359 | Up |
| 3 | P04264 | Keratin, type II cytoskeletal 1 | KRT1 | 2.78017 | Up |
| 4 | P04275 | von Willebrand factor | VWF | 2.39407 | Up |
| 5 | P00739 | Haptoglobin-related protein | HPR | 2.05507 | Up |
| 6 | P22352 | Glutathione peroxidase 3 | GPX3 | 2.04095 | Up |
| 7 | P01023 | Alpha-2-macroglobulin | A2M | 2.00583 | Up |
| 8 | P00918 | Carbonic anhydrase 2 | CA2 | 1.85744 | Up |
| 9 | P05109 | Protein S100-A8 | S100A8 | 1.81789 | Up |
| 10 | Q96IY4 | Carboxypeptidase B2 | CPB2 | 1.56938 | Up |
| 11 | P05546 | Heparin cofactor 2 | SERPIND1 | 1.54135 | Up |
| 12 | P08779 | Keratin, type I cytoskeletal 16 | KRT16 | 1.41883 | Up |
| 13 | P02679 | Fibrinogen gamma chain | FGG | 1.41877 | Up |
| 14 | P35527 | Keratin, type I cytoskeletal 9 | KRT9 | 1.39715 | Up |
| 15 | P07737 | Profilin-1 | PFN1 | 1.23862 | Up |
| 16 | P35542 | Serum amyloid A-4 protein | SAA4 | 1.20988 | Up |
| 17 | P08637 | Low affinity immunoglobulin gamma Fc region receptor III-A | FCGR3A | -1.2344 | Down |
| 18 | Q9Y5Y7 | Lymphatic vessel endothelial hyaluronic acid receptor 1 | LYVE1 | -1.3374 | Down |
| 19 | P05362 | Intercellular adhesion molecule 1 | ICAM1 | -1.3417 | Down |
| 20 | Q02383 | Semenogelin-2 | SEMG2 | -1.3968 | Down |
| 21 | P14174 | Macrophage migration inhibitory factor | MIF | -1.4705 | Down |
| 22 | P04196 | Histidine-rich glycoprotein | HRG | -1.543 | Down |
| 23 | Q9Y6R7 | IgGFc-binding protein | FCGBP | -1.614 | Down |
| 24 | A0A0B4J1V0 | Immunoglobulin heavy variable 3-15 | IGHV3-15 | -1.7064 | Down |
| 25 | Q6UXB8 | Peptidase inhibitor 16 | PI16 | -1.8259 | Down |
| 26 | Q8N6C8 | Leukocyte immunoglobulin-like receptor subfamily A member 3 | LILRA3 | -1.8868 | Down |
| 27 | P17936 | Insulin-like growth factor-binding protein 3 | IGFBP3 | -2.0833 | Down |
