# Supplementary material for: Proteomics and Machine Learning Approaches Reveal a Set of Prognostic Markers for COVID-19 Severity With Drug Repurposing Potential
Source: Front Physiol. 2021 Apr 27;12:652799. doi: 10.3389/fphys.2021.652799 (PMC8120435; doi:10.3389/fphys.2021.652799)
Supplement: Supplementary Table 4 — List of DEPs altered in COVID-19 severe vs. non-severe. [file Table_4.docx]

**Supplementary Table-S4. List of differentially expressed proteins in the COVID-19 Severe versus COVID-19 Non-Severe plasma sample**

| **S.No.** | **ProteinIDs** | **Protein Name** | **Gene** | **Fold Change** | **Trend** |
| --- | --- | --- | --- | --- | --- |
| 1 | P29622 | Kallistatin | SERPINA4 | 3.48822 | Up |
| 2 | P02743 | Serum amyloid P-component | APCS | 3.32873 | Up |
| 3 | P05109 | Protein S100-A8 | S100A8 | 2.90517 | Up |
| 4 | P02679 | Fibrinogen gamma chain | FGG | 2.88435 | Up |
| 5 | P08185 | Corticosteroid-binding globulin | SERPINA6 | 2.85908 | Up |
| 6 | P01011 | Alpha-1-antichymotrypsin | SERPINA3 | 2.59866 | Up |
| 7 | P05543 | Thyroxine-binding globulin | SERPINA7 | 2.37399 | Up |
| 8 | P13796 | Plastin-2 | LCP1 | 2.36932 | Up |
| 9 | P36955 | Pigment epithelium-derived factor | SERPINF1 | 2.3032 | Up |
| 10 | P05546 | Heparin cofactor 2 | SERPIND1 | 2.21335 | Up |
| 11 | P01019 | Angiotensinogen | AGT | 1.69102 | Up |
| 12 | P01023 | Alpha-2-macroglobulin | A2M | 1.60323 | Up |
| 13 | P05155 | Plasma protease C1 inhibitor | SERPING1 | 1.54691 | Up |
| 14 | P00915 | Carbonic anhydrase 1 | CA1 | 1.54499 | Up |
| 15 | P0C0L5 | Complement C4-B | C4B | 1.52731 | Up |
| 16 | P04114 | Apolipoprotein B-100 | APOB | 1.4867 | Up |
| 17 | P06276 | Cholinesterase | BCHE | 1.4702 | Up |
| 18 | P25311 | Zinc-alpha-2-glycoprotein | AZGP1 | 1.45078 | Up |
| 19 | P06310 | Immunoglobulin kappa variable 2-30 | IGKV2-30 | 1.36206 | Up |
| 20 | P22352 | Glutathione peroxidase 3 | GPX3 | 1.29233 | Up |
| 21 | P00746 | Complement factor D | CFD | -1.239 | Down |
| 22 | P08571 | Monocyte differentiation antigen CD14 | CD14 | -1.2672 | Down |
| 23 | P07357 | Complement component C8 alpha chain | C8A | -1.2729 | Down |
| 24 | P08519 | Apolipoprotein | LPA | -1.2876 | Down |
| 25 | O95445 | Apolipoprotein M | APOM | -1.3249 | Down |
| 26 | P05156 | Complement factor I | CFI | -1.3424 | Down |
| 27 | Q16610 | Extracellular matrix protein 1 | ECM1 | -1.3733 | Down |
| 28 | P17936 | Insulin-like growth factor-binding protein 3 | IGFBP3 | -1.3835 | Down |
| 29 | P16070 | CD44 antigen | CD44 | -1.3837 | Down |
| 30 | P18065 | Insulin-like growth factor-binding protein 2 | IGFBP2 | -1.3953 | Down |
| 31 | Q01082 | Spectrin beta chain, non-erythrocytic 1 | SPTBN1 | -1.4009 | Down |
| 32 | Q8N6C8 | Leukocyte immunoglobulin-like receptor subfamily A member 3 | LILRA3 | -1.4206 | Down |
| 33 | P27918 | Properdin | CFP | -1.4917 | Down |
| 34 | P23142 | Fibulin-1 | FBLN1 | -1.5621 | Down |
| 35 | P02652 | Apolipoprotein A-II | APOA2 | -1.7301 | Down |
| 36 | P67936 | Tropomyosin alpha-4 chain | TPM4 | -1.9442 | Down |
| 37 | Q02383 | Semenogelin-2 | SEMG2 | -2.3878 | Down |
| 38 | Q6UXB8 | Peptidase inhibitor 16 | PI16 | -2.4545 | Down |
