# Supplementary material for: Proteomics and Machine Learning Approaches Reveal a Set of Prognostic Markers for COVID-19 Severity With Drug Repurposing Potential
Source: Front Physiol. 2021 Apr 27;12:652799. doi: 10.3389/fphys.2021.652799 (PMC8120435; doi:10.3389/fphys.2021.652799)
Supplement: Supplementary Table 7 — List of proteins involved in the enriched pathways. [file Table_7.docx]

**Supplementary Table-S-7. List of proteins expressed in enrichment pathways**

| **S.No.** | **Biological Pathway** | **GO term Identifier** | **LogP** | **Log**  **(q-value)** | **Proteins involved in pathway** |
| --- | --- | --- | --- | --- | --- |
| 1 | Regulation of peptidase activity | GO:0052547 | -18.794 | -14.448 | A2M, SERPINA3, AGT, SERPING1, C4B, SERPINA6, CD44, ECM1, FBLN1, SERPIND1, SERPINF1, SERPINA4, S100A8, SEMG2, SERPINA7, PI16, APCS, APOA2 |
| 2 | Regulated exocytosis | GO:0045055 | -9.7517 | -6.610 | A2M, SERPINA3, SERPING1, CD14, CD44, CFD, ECM1, FGG, CFP, SERPINA4, S100A8, LILRA3, APOB, SERPIND1, APCS, FBLN1, LCP1, SEMG2, SERPINF1 |
| 3 | Extracellular structure organization | GO:0043062 | -8.3061 | -5.363 | A2M, AGT, APOA2, APOB, CD44, FBLN1, FGG, LCP1, APOM, ECM1, IGFBP2, IGFBP3 |
| 4 | Blood coagulation, fibrin clot formation | GO:0072378 | -7.3361 | -4.521 | A2M, SERPING1, FBLN1, FGG |
| 5 | Complement activation, classical pathway | GO:0006958 | -5.9984 | -3.333 | APCS, SERPING1, C4B, C8A, CFI |
| 6 | Leukocyte activation involved in immune response | GO:0002366 | -5.5396 | -2.942 | SERPINA3, CD14, CD44,CFD, LCP1, CFP, S100A8, LILRA3 |
| 7 | Response to glucocorticoid | GO:0051384 | -4.4234 | -1.996 | APOA2,BCHE,IGFBP2,SERPINF1,AGT,SERPING1,APOB,FBLN1 |
